# Dissection of the Complex Phenotype in Cuticular Mutants of Arabidopsis Reveals a Role of SERRATE as a Mediator

**DOI:** 10.1371/journal.pgen.1000703

**Published:** 2009-10-30

**Authors:** Derry Voisin, Christiane Nawrath, Sergey Kurdyukov, Rochus B. Franke, José J. Reina-Pinto, Nadia Efremova, Isa Will, Lukas Schreiber, Alexander Yephremov

**Affiliations:** 1 Molekulare Pflanzengenetik, Max-Planck-Institut für Züchtungsforschung, Köln, Germany; 2 Department of Plant Molecular Biology, University of Lausanne, Lausanne, Switzerland; 3 Institut für Zelluläre and Molekulare Botanik, Universität Bonn, Bonn, Germany; The University of North Carolina at Chapel Hill, United States of America

## Abstract

Mutations in *LACERATA* (*LCR*), *FIDDLEHEAD* (*FDH*), and *BODYGUARD* (*BDG*) cause a complex developmental syndrome that is consistent with an important role for these Arabidopsis genes in cuticle biogenesis. The genesis of their pleiotropic phenotypes is, however, poorly understood. We provide evidence that neither distorted depositions of cutin, nor deficiencies in the chemical composition of cuticular lipids, account for these features, instead suggesting that the mutants alleviate the functional disorder of the cuticle by reinforcing their defenses. To better understand how plants adapt to these mutations, we performed a genome-wide gene expression analysis. We found that apparent compensatory transcriptional responses in these mutants involve the induction of wax, cutin, cell wall, and defense genes. To gain greater insight into the mechanism by which cuticular mutations trigger this response in the plants, we performed an overlap meta-analysis, which is termed MASTA (MicroArray overlap Search Tool and Analysis), of differentially expressed genes. This suggested that different cell integrity pathways are recruited in *cesA* cellulose synthase and cuticular mutants. Using MASTA for an *in silico* suppressor/enhancer screen, we identified *SERRATE* (*SE*), which encodes a protein of RNA–processing multi-protein complexes, as a likely enhancer. In confirmation of this notion, the *se lcr* and *se bdg* double mutants eradicate severe leaf deformations as well as the organ fusions that are typical of *lcr* and *bdg* and other cuticular mutants. Also, *lcr* does not confer resistance to *Botrytis cinerea* in a *se* mutant background. We propose that there is a role for SERRATE-mediated RNA signaling in the cuticle integrity pathway.

## Author Summary

As the skin of a plant, the epidermis mediates a broad set of protective functions which includes defense against abiotic environmental stresses and pathogens. The majority of its barrier capacity is localized to the outermost cell wall, which is covered by a waxy cuticle. Several distinct cuticular mutants in the model plant Arabidopsis produce a remarkable syndrome that is characterized by ectopic cell adhesion and changes in plant morphology. We used these mutants to study the constitution of the cuticle and the activation of the molecular compensatory mechanisms that are important for adaptation. We examined whole-genome responses in these mutants and used an appropriate statistical procedure to reveal the genes which change their expression. We then applied the same approach to the analysis of hundreds of datasets in repositories. The comparison of gene expression profiles identified the gene *SERRATE*, which encodes a protein of RNA–processing multi-protein complexes, and further analysis revealed that the syndrome is suppressed in double mutants, as predicted. Our finding suggests that the mechanism which operates to control the integrity of the cuticle involves the regulation of small–RNA signaling.

## Introduction

The ability to maintain the barrier properties of the epidermis, which covers the aerial surface of higher plants, is largely due to their outermost cell walls which are impregnated and covered with specialized lipids. The fine structure and composition of this complex layer, called the cuticle, has been the subject of numerous studies [1],[2].

The innermost periclinal layer of the cuticle is, in fact, a cutinized portion of the epidermal cell wall, in which cell wall polysaccharides are perhaps cross-linked to phenolics and aliphatic components of the cutin. The presence of phenolics, which may contribute to the barrier function, is evident in this layer through fluorescence microscopy and chemical analysis [3]. Under transmission electron microscopy, however, this cutinized layer of the cell wall is often heterogeneous in appearance and penetrated by tufts of fibrillar material, or is sometimes barely visible. This is in contrast to the opaque stripe of the continuous cuticle proper which, at a higher resolution, often appears to be finely lamellated; it is composed of polyester cutin and non-hydrolyzable polymer cutan and is, essentially, free of cell wall polysaccharides. In the early stages, in particular, it may have a pectinaceous under-layer. Wax forms the outermost structural layer, although a certain amount of it (intracuticular wax) permeates the interior of the cuticle. Individual plant species display some variations of this standard pattern.

Waxes and cutin represent two groups of cuticular lipids that are supposed to be primarily responsible for the barrier function of the plant epidermis. The structural, biochemical, biophysical and molecular genetic aspects of cuticular lipids and their role in the defense against pathogens have been reviewed elsewhere [2], [4]–[10].

Plant growth and development demand that the cuticle changes continuously and also maintains the balance between rigidity and flexibility. During the early stages of epidermal development, cells are covered with osmiophilic amorphous procuticle, but the lamellar structure of the cuticle proper and the reticulate fibrillar pattern of the cutinized portion of the cell wall may become distinguishable as the cuticle forms [2]. Chemical and structural changes in the cuticle may be beneficial, both in terms of the adaptation to fluctuating environmental conditions and in response to various stresses. However, this view of the cuticle as a dynamic structure has yet to be supported by extensive data.

Recent molecular, genetic studies of cuticular mutants have lead to the identification and characterization of a number of genes involved in various aspects of cuticle formation (reviewed in [5],[6],[9],[11]).

A group of Arabidopsis mutants, including *fiddlehead* (*fdh*), *lacerata* (*lcr*) and *bodyguard* (*bdg*), which are thought to be defective in the biosynthesis of cuticular polyesters, reveals secondary phenotypes which include drastic changes in cell differentiation, plant architecture, organ morphology, pathogen resistance and other elements. This suggests that there exists a pathway that is not only essential for cuticle formation, but may, directly or indirectly, control various cellular processes as well [9]. One of these is the adhesive interaction between the epidermal cells of different organs [12],[13]. No mechanism has yet been demonstrated which might account for the association between the series of phenotypes in the cuticular mutants.

Studies of one of these mutants, *bdg*, which exhibits defects that are characteristic of the loss of cuticle structure, paradoxically revealed that this cuticular mutation accumulates significantly more cutin monomers in the residual cell-wall bound lipids, and more wax [14]. It was, therefore, suggested that plants are capable of repairing cuticular perturbations and re-establishing cuticle homeostasis [14]. *Fdh* and *lcr*, like *bdg*, belong to a class of cuticular mutants that are characterized by secondary phenotypes which include misshapen cells and organs and epidermal fusions [14]–[16]. Their cuticular phenotypes were not, however, examined in detail.

Herein, we report that both *lcr* and *fdh* display an increase in cutin and wax constituents and provide insight into the structural aspects of their cuticle. Using a microarray-based transcriptome analysis, we demonstrate the transcriptional upregulation of wax, cutin, and cell wall and defense genes in *lcr*, *bdg* and *fdh*. We propose that this is a compensatory adaptive response, representing a part of a cell-wall integrity maintenance mechanism. To compare the responses induced by mutations in cuticular and cellulose synthase genes, we used the meta-analytical method MASTA (MicroArray overlap Search Tool and Analysis), which has recently been developed in our lab. When utilizing it for *in silico* suppressor/enhancer screens, we identified *SERRATE* and confirmed that it is required for organ fusions in cuticular mutants. This raises the interesting possibility that there may be a connection between the cuticle formation and morphogenesis.

## Results

### Defining the cuticular phenotype of *lcr*

**Figure 1:**
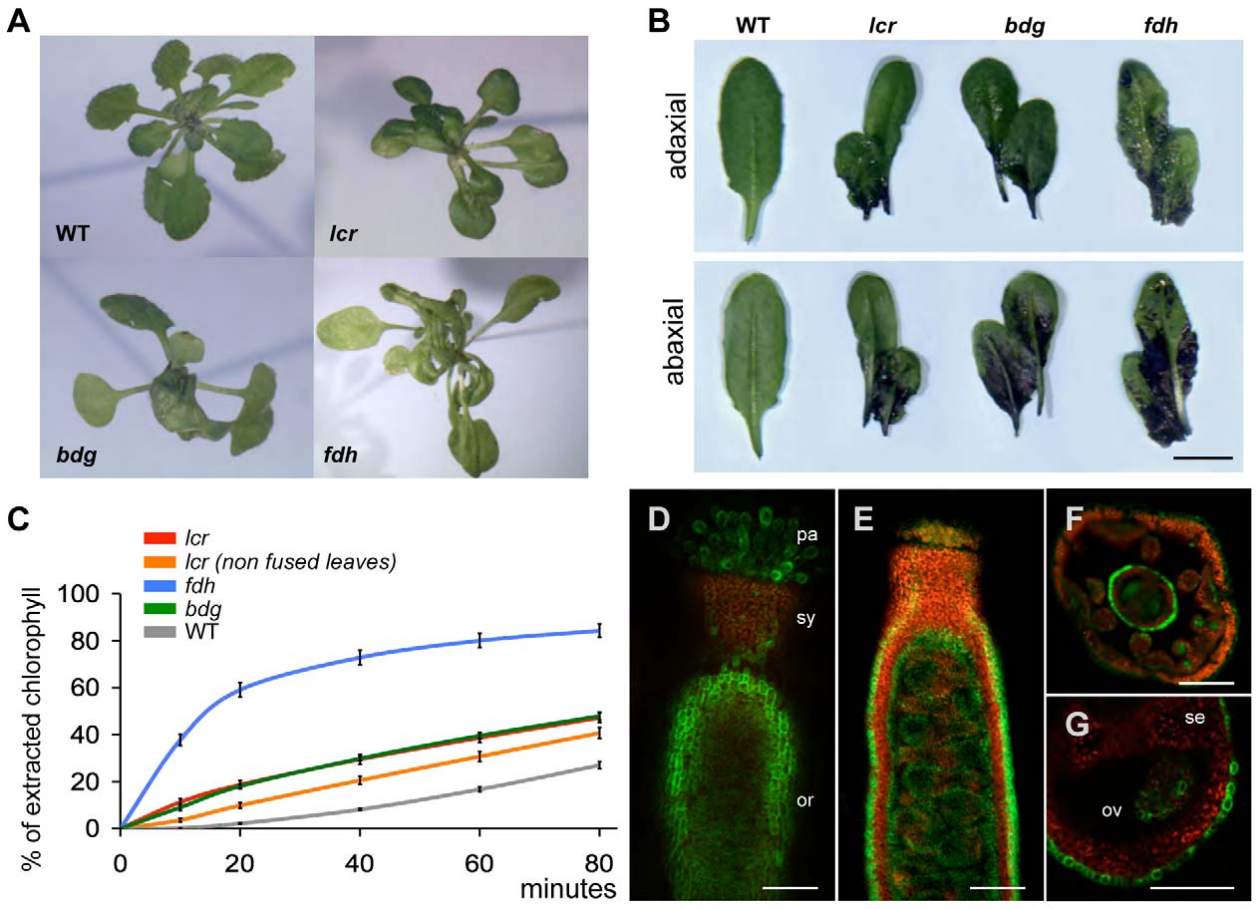
Functional implications and consequences of mutations in *LCR*, *BDG*, and *FDH*. (A) Three mutants, *lcr*, *bdg* and *fdh*, exhibit quite similar organ fusion phenotypes at the rosette stage. Note the differences between the serration of the wild-type leaves and those of the mutants. Leaf deformations in these mutants are not always an after-effect of a fusion at the early developmental stage. (B) Toluidine blue test in *lcr*, *bdg* and *fdh*. Wild-type and mutant leaves are shown after 2 min immersion in a 0.05% toluidine blue solution and destaining in water. No staining is observed with the wild type control. Bar is 1 cm. (C) Chlorophyll leaching test in *lcr*, *bdg* and *fdh*. Typical rosette leaves (∼ten) from seven-eight week old plants (short day conditions) were combined to both compose a sample and spectrophotometrically measure the rate of chlorophyll extraction into an ethanolic solution. The results are mean % values±standard error of at least six replicates. (D–G) Expression of the *LCR*:GFP reporter in transgenic Arabidopsis plants. Shown are the floral organs in which *LCR*:GFP expression is most conspicuous. The green color in the dual-color fluorescent confocal images corresponds to the GFP signal, and the red color to the autofluorescence of the chlorophyll. (D) The pistil, composed of the ovary (or), style (sy), and stigma covered with elongated papilla cells (pa). (E) Optical section through a pistil exposing the interior of the ovary. Note the epidermis-specific expression of GFP in the ovary wall. (F) Cross-section through the floral bud revealing epidermal GFP expression in sepals, petals, stamens and the pistil, and the GFP signal in developing pollen. The pistil epidermis exhibits a brighter signal. (G) Cross-section of the pistil showing septum (se), ovule (ov) and ovary wall in greater detail. Note that the inner epidermis of the ovary wall and the septum are devoid of the GFP signal. Bars are 200 µm for (D,E,F) and 100 µm for (G).

As do other cuticular mutations of this kind, *lcr* has a pleiotropic effect on plant development, which affects leaf morphology, cell morphogenesis and differentiation, shoot branching, and senescence [16]. At the rosette stage, *lcr* plants are easily distinguishable from wild types, but not from *bdg* and *fdh* (Figure 1A), by severe deformations of leaves and leaf fusions. When compared to wild type, the staining of rosette leaves with the water-soluble dye toluidine blue (TB) resulted in *fdh*, *lcr*, and *bdg* having heterogeneous, patchy patterns (Figure 1B), showing the defects of the cuticle. However, with regard to the intensity of staining, neither the fused or unfused rosette leaves of *fdh*, *lcr* and *bdg* were distinguishable from each other. As direct estimation of the cuticle permeability is not feasible in Arabidopsis, to extend these results, we performed an assay which measures chlorophyll leaching into alcohol [13]. As expected, the leaves of all three mutants lost chlorophyll faster than wild type when immersed in 80% ethanol (Figure 1C), thus corroborating the results of the TB staining. However, whereas *lcr* and *bdg* appeared to be very similar to each other, *fdh* released chlorophyll much more quickly (Figure 1C): after 20 min of incubation, *fdh* lost about 60% of total chlorophyll, while *lcr* and *bdg* only lost about 20%.

To study whether there is both a correlation between cuticle permeability to chlorophyll and engagement in ectopic organ fusions, in this assay we examined a sample taken from *lcr* rosette leaves which were not joined in a fusion. Figure 1C shows that these leaves lose the pigment faster than the wild type control does, but this was still slower than the representative *lcr* sample (comprising leaves joined in a fusion and leaves not joined in a fusion). This suggests that both features of the polymorphic *lcr* phenotype are linked.

To investigate whether the expression of *LCR* is restricted to the epidermis, we fused the putative 5′ regulatory regions of the *LCR* gene with the green fluorescent protein (GFP) reporter gene. The expression of *LCR*:GFP was then studied in transgenic Arabidopsis plants by confocal scanning laser microscopy, and was found to be limited to the epidermal cells of leaves, stems, sepals, petals, style, stigma and ovules (Figure 1D–1G). The expression of *LCR* in organ primordia was reminiscent of that of *FDH* and *BDG*, which have previously been studied in detail by using GFP fusions [14],[17]. Because *LCR* belongs to the CYP86A P450 gene subfamily, which includes closely related and highly conserved gene sequences in Arabidopsis, we were not able to design an *LCR* specific probe which would be long enough. We attempted to concatenate *LCR* specific sequence motifs, but the resulting probes also failed to yield a consistent *in situ* hybridization signal (data not shown). However, our results with the *LCR*:GFP plants support the microarray hybridization data which had suggested that *LCR* might be the epidermis specific gene in the stem [18]. Collectively, these results back-up the contention that *lcr* is a typical cuticular gene.

### Ultrastructural changes in the cuticle of *lcr* are reminiscent of that in *bdg* and cutinase expressing (CUTE) plants

**Figure 2:**
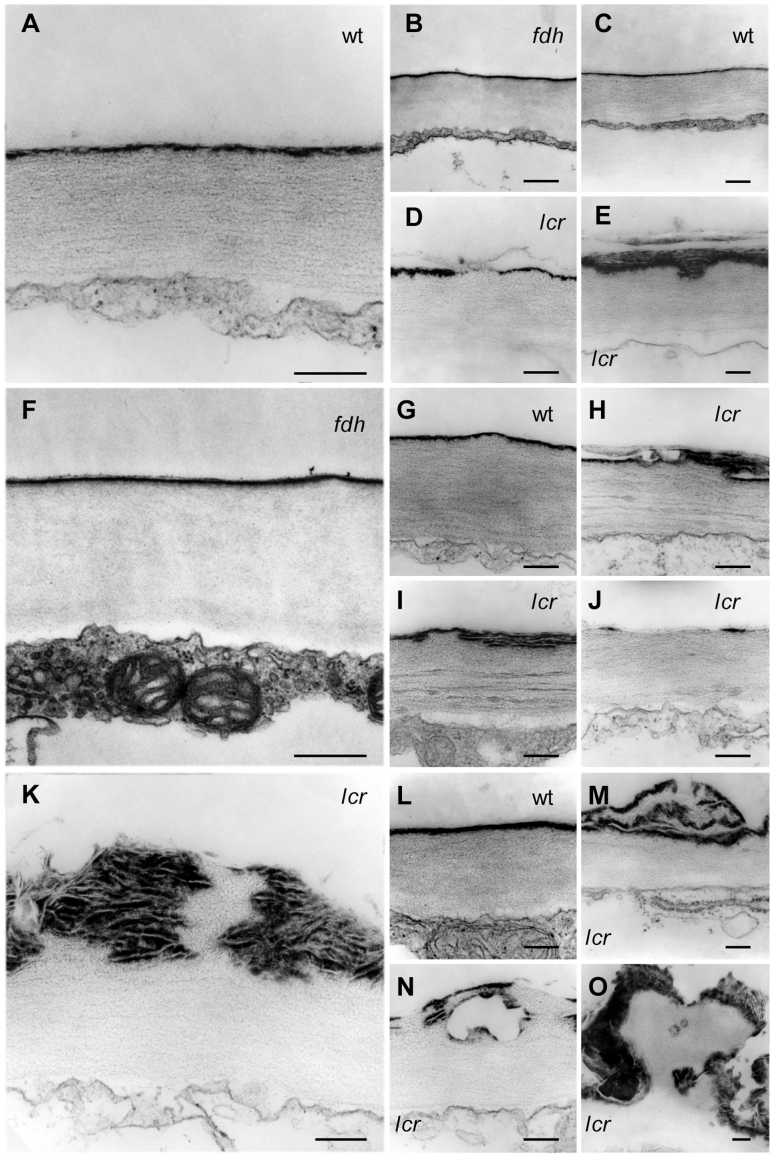
Ultrastructural aspects of the cuticle membrane in *lcr* and *fdh* rosette leaves. The tissues were embedded, and ultrathin sections were stained, and examined through transmission electron microscopy (TEM). Plant types are indicated. (A–E) Young leaves. Adaxial (A,B,D) and abaxial (C,E) cuticle. (F–O) Adult leaves. Adaxial (F,G,H,I,J) and abaxial (K,L,M,N,O) cuticle. Note that the regular electron-dense cuticle proper in both the wild type (wt) and *fdh* are structurally distinct from that in *lcr*. In *lcr*, the cuticle is characterized by depositions of an electron dense crystalloid material, multiple cutin layers, cavities inside the cell wall and breaches at the surface; (O) depicts a notable solid shape bulging out of the cell wall in *lcr*. Bars are 200 nm.

To determine whether the *lcr* mutation distorts or disrupts the cuticle, we examined the epidermis of aerial organs in *lcr* by transmission electron microscopy (TEM). In leaves and petioles of wild type plants, cutin deposition in the epidermal cell wall forms a regular membranous structure (called the cuticle proper) on the outer side (Figure 2A, 2C, 2G, 2L). When viewed under TEM, this electron-dense layer was not only discontinuous and deformed in *lcr*, but was also characterized by the irregular deposition of multi-layered, electron-dense, sharply outlined material, as well as the presence of empty spaces within the deeper layers of the cell wall (Figure 2D, 2E, 2H–2K, 2M–2O). The presence of empty cavities (Figure 2H and 2N) and the over-deposition of an electron-dense material close to the cell wall surface (Figure 2D, 2H, 2K), indicate infiltration, or bursting, of the cell wall materials through the defective cuticle proper in this mutant (Figure 2O), thereby leading to cutin juxtaposition in the inner layers (Figure 2H, 2M, 2N). Supernumerary layers of cutin-like materials may lead to the conclusion that *lcr* does not suffer from a lack of cutin, but rather from the structural dysfunction of its cuticle, even though a cutin overlay was very thin in many instances (Figure 2D, 2K, 2N). In some cases, electron-opaque material seemed to crystallize inside the *lcr* cell wall, giving its cuticle a composite appearance (Figure 2E, 2K, 2O). Although many features make the *lcr* cuticle resemble that in *bdg* [14] and the CUTE plants [19], this has not yet been observed in any other mutant, and appears to be characteristic of *lcr*, which exhibited an extraordinarily irregular cuticle.

In various fusion zones in *lcr*, the cell walls often seemed to be merged, with little or no trace of the intrinsic cuticular membrane, although some inclusions of electron-dense material could be found in areas where cell walls have not been completely fused (Figure S1A, S1B). When exposed to mechanical tension, fused organs might separate, only remaining connected by a fine thread (Figure S1C).

The outer cell wall of epidermal cells in *lcr* was, generally, not as regular as in wild type plants, with severe deformations and some darker stripes giving it a plastic appearance (Figure 2H–2J). This leads to speculation that *lcr* has a cell wall phenotype.

### Ultrastructure of the *fdh* cuticle appears normal in every aspect as evidenced by TEM

From the results of the chlorophyll leaching measurements, it might have been expected that *fdh* would display a highly disorganized cuticle, resembling that in *lcr*. However, Lolle and co-workers reported that the cuticle could always be detected in the fusion zones separating different organs, and three lipid stains failed to detect any differences between *fdh-1* and wild-type tissues [12],[13]. The *fdh-1* allele has been isolated in the Landsberg *erecta* (L*er*) genetic background. Since the *lcr* transposon insertion allele used in this study was found in the Columbia (Col) ecotype, we sought to examine the cuticle in an *fdh* allele with the same genetic background. We made the decision to characterize *fdh-3940S1* [20], which has also been used in the chlorophyll leaching assays described above.

The extensive investigation under TEM did not reveal any visible ultrastructural changes in the *fdh-3940S1* cuticle (hereafter referred to as *fdh*) in different organs when compared to wild-type. This makes *fdh* different to the *lcr*, *bdg* and *hth* mutants and the CUTE plants [19]. A typical, continuous electron-dense layer was found to be deposited in the epidermal cell wall in *fdh* leaves (Figure 2B, 2F) and in the fusion zones (Figure S1), showing that two knock-out alleles of *fdh* behave similarly in the L*er* and Col genetic backgrounds. While the cuticle proper in *fdh* showed no major ultrastructural changes that were detectable by TEM, some images gave the impression that its surface may have a rather more diffused appearance, lacking sharp margins.

These results imply that structural defects in the cuticle proper, which are detectable under TEM, may not account for the molecular sieving properties of the cuticle that were estimated by molecular leaching assays. This also suggests that the organ fusions observed in *fdh*, *lcr*, *bdg* and some other cuticular mutants are not a direct consequence of major structural changes in their cuticles, thereby calling into question the conventional view that the bare cell walls of epidermal cells interact to produce a fusion.

### Resistance to *Botrytis cinerea* in cuticular mutants

**Figure 3:**
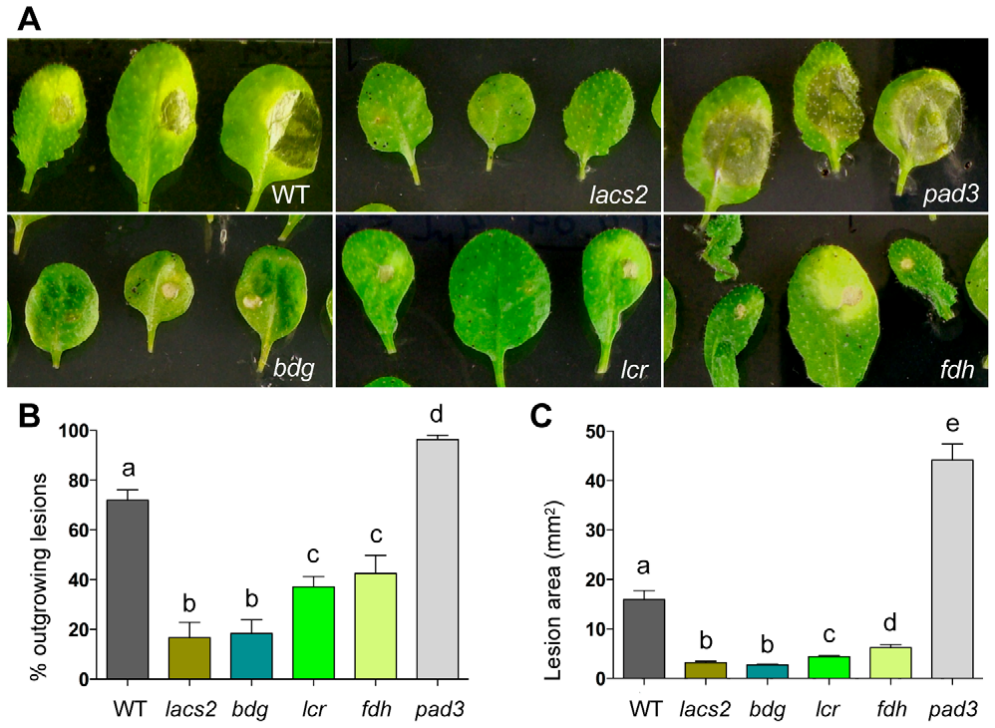
Resistance of cuticular mutants to *Botrytis cinerea*. (A) The appearance of leaf symptoms. Detached leaves were placed on the agar media in Petri dishes, droplet-inoculated with conidiospores of *B. cinerea* strain 2100 and examined at 3 dpi. Three representative leaves are shown per genotype. (B) Percentage of outgrowing lesions at 3 dpi (mean±se). At least 50 leaves were used per genotype, and the statistical significance was calculated in comparison to inoculated wild type using Fisher's exact test. Different letters indicate significance at P<0.01. All differences between mutants and wild type were significant at the P<0.001; *lcr* and *fdh* are more susceptible than *lacs2* and *bdg* at P<0.05. (C) Surface lesion areas (mean±se) for the same samples shown in (A) and (B). Genotypes assigned the same letters are statistically similar to each other at P<0.05.

One of the cuticle's primary roles is to act as a protective barrier against pathogen attack [10]. It has, however, been reported that some Arabidopsis cuticular mutants, such as *lacs2*, *lcr*, *bdg* and transgenic CUTE plants, are, in fact, more resistant to the major necrotroph *Botrytis cinerea* than the wild type [21]–[23]. The reasons for this paradoxical resistance remained unclear. The easier diffusion of a plant-produced toxin through the mutant cuticles was, however, considered to be one of the possible mechanisms of this resistance [21], suggesting that the highly permeable cuticle would greatly increase the resistance of *fdh* to this pathogen. To test this concept, we compared the wild type and three mutants by the detached-leaf assay which appeared to have the best consistency [24]. As controls, we used the *B. cinerea*-resistant *lacs2* and the hypersusceptible *phytoalexin deficient3* (*pad3*) which is impaired in the accumulation of the antifungal metabolite camalexin [25]. The level of susceptibility after droplet inoculation was measured as the percentage of lesions larger than the original inoculation site (percentage of outgrowing lesions) and as the average lesion area (Figure 3). Three days post-inoculation (dpi) with *B. cinerea* (strain 2100 in 1/4 PDB), *lacs2* and *bdg* showed lower susceptibility (P<0.0001), with 16% and 18% of outgrowing lesions, respectively, as compared to 72% in the wild type, and 37% and 42% in *lcr* and *fdh*, respectively (Figure 3B). Consistent with the described hypersusceptibility to *B. cinerea*, 96% of outgrowing lesions were identified in *pad3* (Figure 3B). The higher resistance phenotype of cuticular mutants was revealed by both the lower proportion of outgrowing lesions and lesion area, the latter being on average very similar in all cuticular mutants and always significantly smaller when compared to the wild type (Figure 3C). One interesting observation is that, under our experimental conditions, some leaves of *bdg* and *lacs2* remained free of disease symptoms at 3 dpi (∼27% and ∼56% respectively), whereas all inoculated leaves of *lcr* and *fdh* showed signs of fungal infection. Comparable results were obtained with the *B. cinerea* strain B05.10 (data not shown), corroborating the view that FDH deficiency afforded similar protection against *B. cinerea* as that observed in other cuticular mutants. However *fdh* does not seem to be more resistant than *bdg* and *lcr*, implying that the resistance phenotype does not correlate well with the cuticular permeability when measured by the chlorophyll leaching rate and the TB stainability.

### *Lcr* and *fdh* over-accumulate major cutin monomers

Although *lcr* and *fdh* were recognized as classical cuticular mutants revealing enhanced epidermal permeability (Figure 1B and 1C) [13],[16],[21],[26], the chemical compositions of their cuticular lipids had not been characterized in detail. LCR, which functions as a fatty acid ω-hydroxylase when expressed in yeast, was proposed as being active in the biosynthesis of the ω-hydroxy and α,ω-dicarboxy fatty acids that are major cutin monomers in Arabidopsis [16],[27]. The loss of the LCR function is presumed to reduce the accumulation of the respective cutin monomers.

Based on the amino acid sequence similarity, it has been suggested that *FDH* encodes a β-ketoacyl-CoA synthase (KCS) which is involved in microsomal fatty acid elongation [20],[28]. In *FDH*-deficient plants, cutin monomers could, therefore, potentially either decrease or comprise shorter chain monocarboxylic and dicarboxylic fatty acids.

**Figure 4:**
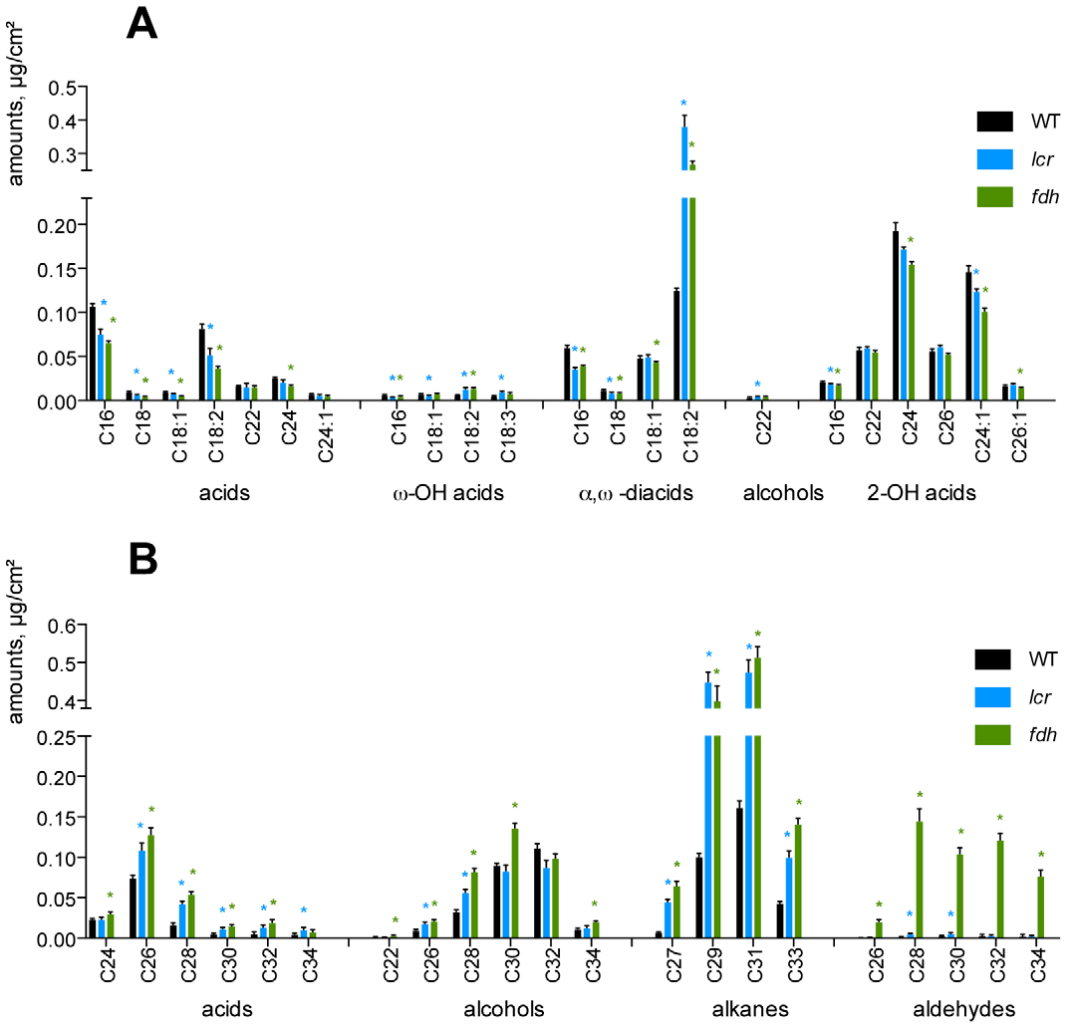
Analysis of leaf residual-bound lipids and wax in *fdh* and *lcr*. (A) The fatty acid composition analysis of the leaf residual bound lipids that are left after exhaustive extraction with methanol/chloroform. Values are mean±standard error for five (*lcr*), six (wild type) or seven (*fdh*) replicates, each containing leaves from at least 15 plants. (B) The composition of the analysis of leaf wax. Values are mean±standard error from six (*fdh*) or seven (wild type and *lcr*) replicates, each containing leaves from at least ten plants. Stars indicate in (A) and (B) a significant Mann-Whitney test (two-tailed, P<0.05) for mutant versus wild type.

To determine the effects of *fdh* and *lcr* mutations on the chemical composition of cuticular polyesters, we analyzed residual-bound lipids in leaves (Figure 4A). This approach gives a good approximation of the monomer composition of pure cutin, which is difficult to isolate in sufficient amounts in Arabidopsis [29]. This analysis was conducted twice on both mutants, using plant material grown under similar greenhouse conditions in different seasons (Figure 4A and Figure S2). Both experiments yielded similar results, with *lcr* and *fdh* accumulating higher levels of the C18:2 α,ω-diacid which is a major cutin component in Arabidopsis [29],[30]. The increase, when compared to wild type, was approximately three times and twice in *lcr* and *fdh*, respectively (Figure 4A). Remarkably, the levels of C18:2 ω-hydroxy acid, which is a precursor to C18:2 α,ω-diacid, also increased two-fold in both mutants.

Both two and one and half times differences were also found for the C18:3 ω-hydroxy acid in *lcr* and *fdh*, respectively. It is also worth noting that both accumulated approximately less than twice the C18:2 acid in residual-bound lipids. The partially (by ∼25–40%) reduced content of the C16:0 acyl chains in the three classes of fatty monomers (acids, ω-hydroxy acids, α,ω-diacids), might be evidence of an enhanced C16 elongation in the mutants.

Examination of the cutin composition analysis data (Figure 4A and Figure S2) revealed that no shift towards shorter carbon chains could be detected in *fdh*. Moreover, no consistent decrease in the levels of ω-hydroxy fatty acids and α,ω-diacids could be detected in *lcr*. Given the increase in the levels of C18 ω-hydroxy fatty acids and α,ω-diacids, it could be proposed that the major changes in cuticular lipids observed in *lcr* and *fdh* are not due to the deficiency caused by the respective mutations. Instead, it could be evidence of an induced response to these mutations, which leads to the incorporation of more cutin-like material in the outer epidermal cell wall of the mutants. This response could play a compensatory role which contributes to the survival of the mutant plants. It is particularly noteworthy in this context that *lcr* and *fdh* appear to possess remarkably similar cutin monomer compositions.

### The *lcr* and *fdh* mutations lead to an over-accumulation of cuticular wax

There is a strong line of evidence linking the over-accumulation of epi- and intra-cuticular wax to the cuticular deficiency in *bdg* [14]. We, therefore, analyzed leaf wax composition in *lcr* and *fdh* (Figure 4B), revealing that the total amount of wax had been increased two-fold and three-fold in *lcr* and *fdh*, respectively; on average, wild-type leaves had a wax load of 0.72±0.07 µg/cm^2^ compared to 1.56±0.25 µg/cm^2^ and 2.66±0.25 µg/cm^2^ in *lcr* and *fdh*, again respectively. The major constituents of wax, which are alkanes in Arabidopsis, were increased from 2.3 times (C33 in *lcr*) to 9 times (C27 in *fdh*). The levels of free fatty acids and alcohols had also increased, but to a lesser extent, with up to a 3.3-fold increase in the C32 fatty acid (Figure 4B). The marked difference between the two mutants was the extent to which wax aldehydes were produced. Whereas these had not changed in *lcr* when compared to wild type, the *fdh* leaf wax appeared to contain much greater amounts of all aldehyde species than the wild type did, with C28 exhibiting the biggest (nearly a 830-fold) increase.

We also examined the leaf epidermis in *lcr*, *fdh*, *bdg* and wild type by cryo-scanning electron microscopy (SEM) which preserves wax morphology. Under SEM, the leaf surface in the wild type appeared to be smooth, without appreciable sculpturing. However, in all three mutants examined, a considerable number of ripples and plate-like wax crystals gave their surfaces a more ruffled appearance (Figure S3).

We concluded from these results that *lcr* and *fdh* respond to the loss of respective gene functions by the over-accumulation of wax in leaves. This response is quite similar with respect to alkanes, but only *fdh* appears to activate a pathway for aldehyde biosynthesis. Our SEM results suggest that stem wax is also affected in these mutants (Figure S4), but a detailed analysis would be beyond the scope of this paper.

### Genome-wide analysis shows coordinated differential gene expression in three cuticular mutants

The cuticular phenotypes of *lcr* and *fdh* described above, and the analysis of their cuticular lipids, suggest that these mutations may induce a kind of a response which includes the genes associated with cutin and wax biosynthesis. If this response is controlled at the level of transcription or mRNA stability, coordinated changes in the abundance of transcripts which encode corresponding genes should be observed in the cuticular mutants. To test this possibility, we studied the gene expression changes by using microarray hybridization with the Arabidopsis ATH1 Genome Array (Affymetrix). We then compared gene expression in young rosette leaves from *fdh*, *lcr* and *bdg* mutants to that in wild type. As described in Materials and Methods, RNA-derived probes were prepared from three biologically independent samples for each mutant.

**Figure 5:**
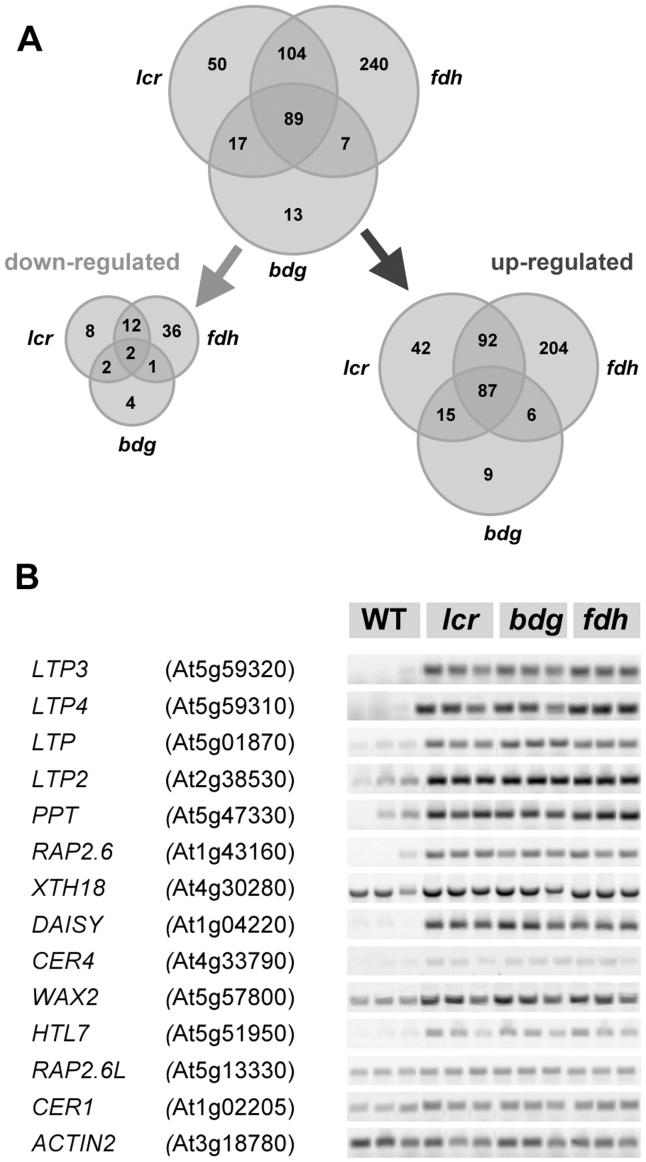
An overview of the microarray results in the three mutants. (A) Gene categories revealed by Rank Product statistical analysis, followed by a Venn diagram representing overlapping or non-overlapping gene sets. Differentially expressed genes were defined by pfp<0.05 (corresponds to FDR<0.05) between mutant and wild type samples. The set, which comprises 87 upregulated and 2 downregulated genes common to all three mutants, is detailed in Table S3. (B) Confirmation of the microarray data by semi-quantitative RT–PCR. Thirteen candidates were compared to ACTIN2 as a control. To allow the semi-quantitative estimation of differences, the number of PCR cycles has been optimized for each gene.

Taking into account the low replicate numbers of the microarray data, we have chosen to detect differentially expressed genes (DEGs) using the Rank Product (RP) method, as suggested by Breitling and co-workers [31]. This produces a good performance, in particular for replicate numbers below ten [32]. We recently revealed that RP outperforms Cyber-T, Local Pooled Error (LPE), two-sample Bayes T, Empirical Bayes, SAM, fold change and the ordinary t-test in terms of the validity of the DEG lists [33]. The significance of the detection is assessed in RP by a non-parametric permutation test which evaluates the percentage of false positive predictions (pfp) or the false discovery rate (FDR). In this study, we regarded genes with a pfp of less than 5% (0.05) to be significantly differentially expressed because, for them, the probability of being consistently selected by the RP method is greater than 95%. This filtering resulted in a list of 440 DEGs in *fdh* when compared to wild type, followed by *lcr* (260 DEGs) and *bdg* (126 DEGs). The DEGs are listed in Tables S1, S2. Figure 5A summarizes the findings, and shows Venn diagrams which represent the number of genes that were changed and up or downregulated in the three cuticular mutants. The microarray analysis suggests that the majority of DEGs were upregulated: 88% for *fdh*, 91% for *lcr*, and 93% for *bdg* (Figure 5A). It also reveals large overlaps between misregulated genes in the different mutants. This supports the notion that these mutants exhibited similar transcriptional changes, as suggested by similarities in their organ fusion phenotypes and the composition of their cuticular lipids. The expression of only 13 genes (10%) was specifically changed in *bdg*, compared to 50 specific genes (19%) in *lcr* and 240 specific genes (54%) in *fdh* (Figure 5A). Table S3 includes 89 genes which were found in the overlap between the genes that were differentially expressed in all of these mutants. Remarkably, these 89 common genes represent 71% of all of the DEGs in *bdg*. A simplified version of this table is shown in Table 1.

**Table 1:** A subset of common, statistically significant DEGs in three cuticular mutants.

AGI ID	Annotation	*bdg* rank	*bdg* FC	*bdg* pfp	*lcr* rank	*lcr* FC	*lcr* pfp	*fdh* rank	*fdh* FC	*fdh* pfp	V	*se*
	**Up-regulated genes**											
At5g59320	LTP3 (LIPID TRANSFER PROTEIN3)	3	62.9	0.000	2	84.6	0.000	1	172.5	0.000	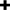	
At5g59310	LTP4 (LIPID TRANSFER PROTEIN4)	2	67.2	0.000	3	67.5	0.000	2	137.9	0.000	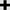	
At5g01870	LTP, lipid transfer protein	1	78.9	0.000	5	45.5	0.000	5	50.2	0.000	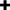	
At2g38530	LTP2 (LIPID TRANSFER PROTEIN2)	4	52.9	0.000	4	58.4	0.000	4	100.7	0.000	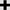	
At1g52690	LEA (late embryogenesis abundant) protein	6	45.8	0.000	1	89.9	0.000	8	50.3	0.000		
At5g47330	PPT, palmitoyl protein thioesterase	9	30.2	0.000	7	36.8	0.000	10	37.3	0.001	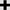	
At2g39350	ABC transporter	10	25.4	0.000	9	23.1	0.000	7	41.2	0.000		
At3g22600	LTP, lipid transfer protein	7	37.3	0.000	11	21.8	0.000	9	48.7	0.000		
At4g30290	XTH19 (XYLOGLUCAN	8	27.1	0.000	13	21.5	0.000	12	32.4	0.001		
	ENDOTRANSGLUCOSYLASE/HYDROLASE19)											
At5g09530	HRGP (hydroxyproline-rich glycoprotein)	5	44.7	0.000	30	12.2	0.001	3	62.2	0.000		
At1g43160	RAP2.6, AP2 domain-containing transcription factor	18	12.8	0.001	6	32.9	0.000	20	24.0	0.001	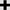	
At2g43620	chitinase	25	10.5	0.001	12	24.1	0.000	13	33.5	0.001		▾
At5g26340	STP13 (SUGAR TRANSPORT PROTEIN13)	31	7.9	0.001	29	13.5	0.001	6	46.7	0.000		
	hexose∶hydrogen symporter											▾
At4g21680	POT (proton-dependent oligopeptide transport) protein	17	12.9	0.001	10	20.4	0.000	44	14.0	0.002		▾
At2g37770	aldo/keto reductase	11	16.4	0.000	26	12.6	0.001	34	15.7	0.002		▾
At1g30720	FAD-binding domain-containing protein (also At1g30730)	43	6.6	0.004	16	18.2	0.001	16	25.6	0.001		▾
At1g75750	GASA1 (GAST1 PROTEIN HOMOLOG1)	16	11.6	0.001	23	12.6	0.001	36	14.8	0.002		
At2g39200	MLO12 (MILDEW RESISTANCE LOCUS O 12)	30	7.9	0.001	24	14.1	0.001	27	16.7	0.002		▾
At4g25810	XTR6/XTH23 (XYLOGLUCAN	22	9.9	0.001	15	21.8	0.001	47	14.0	0.002		▾
	ENDOTRANSGLYCOSYLASE6)											
At5g67480	BT4 (BTB AND TAZ DOMAIN PROTEIN4)	13	17.2	0.000	22	15.2	0.001	54	13.8	0.003		
At5g64120	peroxidase	58	4.9	0.011	21	14.8	0.001	11	31.7	0.001		▾
At3g20470	GRP5 (GLYCINE-RICH PROTEIN5)	15	14.3	0.001	58	7.7	0.006	21	22.4	0.001		
At1g51800	leucine-rich repeat protein kinase	39	7.9	0.003	33	12.6	0.001	23	20.2	0.001		▾
At4g30280	XTH18 (XYLOGLUCAN	46	6.5	0.007	8	24.5	0.000	57	12.9	0.004	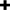	▾
	ENDOTRANSGLUCOSYLASE/HYDROLASE 18)											
At3g45060	NRT2.6 (high affinity nitrate transporter 2.6)	53	5.3	0.010	45	8.9	0.003	22	20.5	0.001		
At1g52890	NAC019, NAC-domain transcription factor	52	6.1	0.008	20	15.2	0.001	49	14.5	0.002		
At3g02480	ABA-responsive protein-related	28	8.6	0.001	17	16.3	0.001	77	11.4	0.004		
At1g21910	AP2 domain-containing transcription factor	54	5.4	0.010	32	11.9	0.001	42	15.1	0.002		
At3g14060	unknown protein	27	9.5	0.001	41	9.8	0.002	61	12.0	0.004		
At5g20230	BCB (BLUE-COPPER-BINDING PROTEIN)	60	4.8	0.011	43	10.3	0.002	32	17.3	0.002		▾
At2g25000	WRKY60, WRKY domain-containing	12	15.1	0.000	62	6.7	0.006	68	11.4	0.004		
	transcription factor											
At4g39670	glycolipid transporter	99	3.9	0.032	37	10.9	0.001	14	28.9	0.001		
At3g57520	ATSIP2 (SEED IMBIBITION 2) hydrolase	73	3.7	0.021	51	8.3	0.003	26	18.3	0.002		
At1g02400	GA2OX6/DTA1 (GIBBERELLIN 2-OXIDASE 6)	42	6.2	0.004	31	11.8	0.001	79	10.8	0.004		
At4g12470	LTP, lipid transfer protein	72	3.9	0.020	56	7.7	0.004	24	20.2	0.001		
At1g02850	glycosyl hydrolase family 1 protein	35	8.2	0.001	54	8.8	0.004	65	12.4	0.004		▾
At4g37990	ELI3 (ELICITOR-ACTIVATED GENE 3)	32	8.7	0.001	36	11.3	0.001	97	9.7	0.006		
At1g07610	MT1C (metallothionein 1C)	14	14.4	0.001	14	20.5	0.000	142	7.8	0.009		
At1g04220	DAISY, beta-ketoacyl-CoA synthase	21	9.0	0.001	83	5.8	0.008	72	11.7	0.004	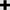	
At5g05600	oxidoreductase	112	3.5	0.046	44	10.9	0.003	30	17.0	0.002		▾
At4g27410	NAC072/RD26 (RESPONSIVE TO DESSICATION 26), NAC-domain transcription factor	41	7.2	0.004	28	13.6	0.001	125	8.7	0.007		▾
3g46280	protein kinase-related	90	3.4	0.031	92	5.6	0.009	18	20.3	0.001		▾

The 42 top upregulated genes out of 89 statistically significant genes are listed here. A full version of this table is provided as Table S3. Rank shows position of each gene in the respective gene list ordered by pfp value; FC (fold change) is calculated as a mutant/wild type ratio; pfp is the probability that a gene identified as having a significantly different expression is, in fact, not significantly different. Plus denotes a positive PCR test (V). A filled in inverted triangle denotes the genes that are downregulated in the *serrate (se)* mutant. The genes in this table were ordered by the sum of ranks.

To substantiate this computational analysis, we re-calculated the microarray data as one experiment which consisted of two groups: the cuticular mutant group with nine replicates and the wild type group with three. This experimental design should minimize the inter-dependence between mutant versus wild type group differences. One would also expect statistical power to increase as the number of replicates goes from three to nine for the mutant group. By using the same parameter settings in the Rank Product method, and the same pfp cut-off value, we obtained a list of 744 upregulated DEGs. Compared to the 87 commonly upregulated genes which were identified with the first approach, many more candidates were detected by Rank Product this time, suggesting that the actual number of genes discriminating between the three cuticular mutants and wild type is higher. However, 74 (85%) of these 87 genes were found in the overlap with the top-87 gene list from the second approach. A comparison of gene ranking also reveals that there is a significant consensus between the lists obtained by the two approaches.

### Verification of microarray hybridization by semi-quantitative RT–PCR and *in situ* hybridization

To further corroborate our results, we sought to first demonstrate by semi-quantitative RT–PCR that the selected genes are, in fact, up or downregulated as predicted by the microarrays. We have chosen 12 candidate genes from among those which are commonly upregulated in the three mutants (Table S3), as well as two genes which were not included in this list. The first was *CER1*, which is thought to be directly involved in wax biosynthesis, although its enzymic function remains unknown; the other was a *RAP2.6*-like gene (*RAP2.6L*) which encodes an AP2/EREBP domain protein (Text S1). *RAP2.6L* was one of the genes which appeared to be upregulated, but did not meet the criteria because one of its pfp values was slightly above the 0.05 cut-off (0.052). From the microarray hybridization analysis, we estimated that the selected genes were upregulated in the range of 3 to 172-fold in the mutants (Table S3).

The results of semi-quantitative RT–PCR assays, shown in Figure 5B, indicated that all selected genes were consistently upregulated in the mutants. Interestingly, one can also observe a correlation between the genes that are strongly upregulated in both assays (namely the microarray and the semi-quantitative RT–PCR) with the *LTPs*, *PPT*, *RAP2.6* and *DAISY* genes which display the most distinct expression between mutants and wild type. However, we did not aim to quantitatively evaluate gene expression measurements, or compare respective fold changes which would have required the use of real-time, quantitative RT–PCR.

One of the genes which was found to be highly up-regulated in the leaves of all three mutants (in *lcr* 5.8-fold, in *fdh* 11.7-fold and in *bdg* 9.0-fold, as revealed by microarrays) was *DAISY* (Table 1). It was shown to encode a KCS which is involved in the biosynthesis of aliphatic suberin in roots. The roots of the *daisy* mutant accumulate significantly less C22 and C24 very-long-chain fatty acid derivatives in suberin, suggesting that it functions as a docosanoic acid synthase [34]. The RT–PCR analysis and promoter-GUS fusions revealed that it was also expressed in various aerial organs of the plant, although its expression levels in leaves were very low [34]. While *DAISY* was almost undetectable in unwounded rosette leaves, its expression was rapidly induced by wounding, and correlated with suberin deposition around punctures [34]. These features render *DAISY* a good target for confirmation by *in situ* hybridization. In particular, we wondered whether it would be specifically induced in the leaf epidermis of the cuticular mutants.

**Figure 6:**
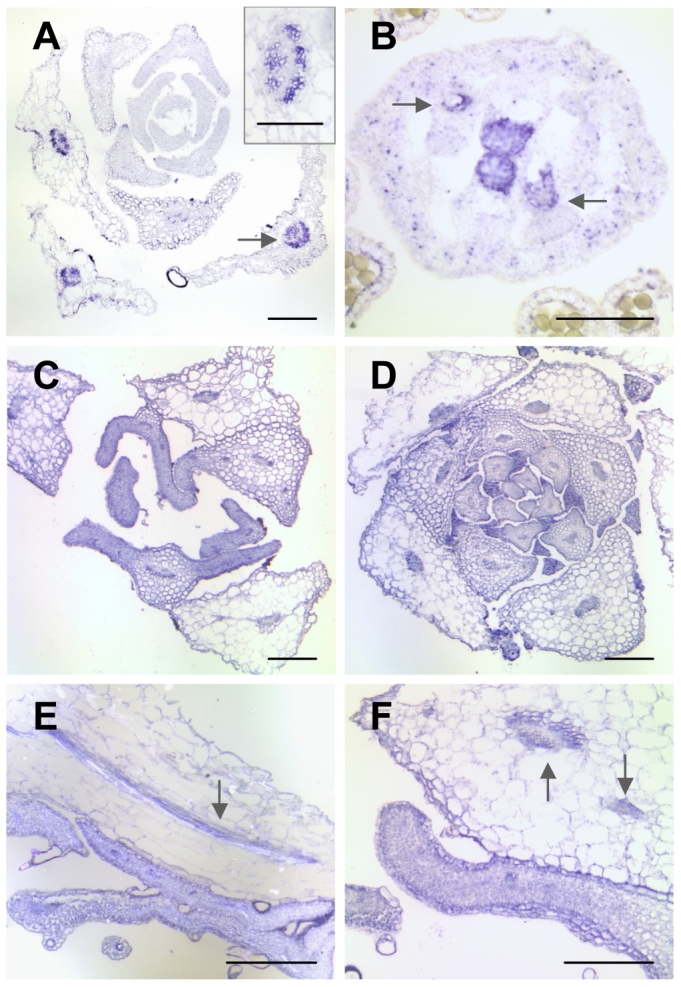
Expression pattern of *DAISY* in the *lcr*, *bdg*, and wild-type plants. The tissue-specific localization of *DAISY* mRNA was revealed by *in situ* hybridization using the antisense riboprobe. (A) Cross-section showing expression in the wild type rosette. Arrow indicates an *in situ* hybridization signal in a vascular bundle of developing leaves. The upper right insert shows a magnified view of a vascular bundle. (B) Cross-section through the stylar tissue of a wild-type pistil, revealing the expression signal in the pollen transmitting tract and ovules (arrows). (C) Cross-section of the *lcr* rosette. (D) Cross-section of the *bdg* rosette. Note that *DAISY* is ectopically induced in young developing leaves. (E) Semi-longitudinal section showing fusions between younger and older leaves in *lcr*. Arrow depicts signals in the vasculature of the older leaf. (F) Cross-section through fusions between younger and older leaves in *bdg*. Arrows depict signals in the vasculature of the older leaf. Note that the hybridization signal (E) and (F) in the younger leaves is comparable to that in the vascular bundles (arrows), and it may be somewhat stronger in the epidermis than in the inner tissues. Bars are 200 µm for (A,C,D,E,F), and 100 µm for (B) and the insert in (A).

The *in situ* hybridization results from wild type leaves, as shown in Figure 6A, demonstrate that the expression of *DAISY* is restricted to the xylem and phloem in vascular bundles of older rosette leaves; the hybridization signal was also observed the transmitting tract and ovules (Figure 6B). In the *lcr* and *bdg* mutants, the signal was also detected in leaf primordia and young developing leaves (Figure 6C–6 F), and the intensity of labeling in these organs was similar to that in the vasculature (Figure 6E and 6F). In the mutants, *DAISY* was ectopically expressed in all cell types in leaves, including the epidermis, and careful examination did not reveal any cell specificity. We concluded that the enlargement of the domain of *DAISY* expression obtained by *in situ* hybridization is in agreement with our microarray data and the results of semi-quantitative RT–PCR, all of which evidence the induction of *DAISY* in young leaves of *lcr* and *bdg*. We also concluded that the data from the microarray hybridization are reliable and suitable for a comparative analysis.

### The range of commonly upregulated genes in the three cuticular mutants suggests a remodeling of the cell wall and the cuticle and activation of defenses to abiotic stresses and pathogens

To find out which biological processes are most affected in cuticular mutants, we first used the Classification SuperViewer [35] which analyses Gene Ontology (GO) annotations (ATH_GO_GOSLIM.20070512) in order to identify overrepresented GO terms when compared to the entire Arabidopsis genome. The most prominent functional group of the upregulated genes (Table S3) was represented by “cell wall” related proteins, with a 15.8±4.7-fold enrichment (mean±standard deviation for 100 bootstraps) when compared to the whole genome. These were followed by the “extracellular” (7.6±3.3), “response to abiotic or biotic stimulus” (3.5±1.0), “plasma membrane” (2.2±1.7), “response to stress” (2.1±0.9), “other enzyme activity” (2.1±0.5), “developmental processes” (1.7±0.6), “hydrolase activity” (1.7±0.5), “transport” (1.7±0.6), and “other membranes” (1.7±0.3) groups. Other listed terms included “electron transport or energy pathways”, “transcription factor activity”, “signal transduction” and “transcription”, but these were less conspicuous. These results strongly indicate that the cell wall undergoes extensive changes in response to cuticular mutations. Given that the cuticle is an essential part of the cell wall, this should not come as a surprise. However, it is interesting that the genes associated with responses to abiotic or biotic stimuli were only third in this list, suggesting that the response to cuticular dysfunction leads to a specific compensatory response whereby the normal homeostasis of the cell wall (and cuticle) is altered, and the viability of the mutants is increased.

Our survey also showed that genes potentially associated with the cell wall, the cuticle and defense responses are upregulated in the cuticular mutants (Text S1). Although the list of commonly misregulated genes presented in Table S3 may not be complete and may contain about 5% of falsely discovered genes, this is a further indication that *bdg*, *lcr* and *fdh* respond similarly to the dysfunction by remodeling their cuticles and cell walls and activating defenses.

### Developing MASTA for the meta-analysis of microarray data

The above findings, when taken together, suggest that the transcriptional activation of target genes is an adaptive response to cuticular mutations such as *bdg*, *lcr* and *fdh*. Moreover, the fact that the three mutants display a peculiar phenotype, which is comprised of the overproduction of wax, organ fusions, irregular leaf shapes and defects in cell differentiation, suggests that the underlying signaling pathways may be distinct from, but overlap with, those that are activated in response to conventional biotic and abiotic factors.

To identify related pathways, we sought to determine which transcriptional responses were the most similar to those observed in *bdg*, *lcr* and *fdh*. We also thought that contrasting these results to a similar analysis for cell wall deficient mutants would help further in the definition of the mechanism by which cuticular mutations induce plant defenses.

To this end, a comparative analysis of differentially regulated genes among the three mutants should be extended to include several hundred of the microarray datasets that are available for Arabidopsis. This is challenging to implement because the generally low consistency of differentially expressed gene (DEG) lists achieved with the use of t-type tests has been reported by several groups, including those participating in the MicroArray Quality Control (MAQC) project [36]. It would be beyond the scope of this paper to go further into the computational details but, using the human MAQC project [36] and the Arabidopsis datasets available from the Gene Expression Omnibus (GEO), we have conducted a comparative survey of the acceptability of several statistical approaches. This revealed that the DEG lists selected by the Rank Product method [31] outperform, in terms of consistency, those generated by seven other methods [33].

Building upon this finding, we developed guidelines for a large-scale comparative analysis of DEG lists, and re-analyzed over 600 contrasts (e.g. mutant vs. wild type or treatment vs. control comparisons) with the Rank Product method, using the raw probe intensity data from the Affymetrix CEL files that we obtained from several databases and authors. We termed this approach MASTA (MicroArray overlap Search Tool and Analysis), after the phrase FASTA that is used for sequence comparison.

### Cell wall biosynthesis mutations and cuticular mutations are likely to induce defense responses through distinct pathways

Mutations in *CELLULOSE SYNTHASE4 (CESA4)/IRREGULAR XYLEM5 (IRX5)* result in modifications in the composition and structure of the secondary cell wall and lead to the specific activation of defense pathways, suggesting that a cell wall integrity system which is similar to that of yeast may exist in plants [37]. This makes the side-by-side meta-comparison with cuticular mutants worthwhile. We, therefore, probed the MASTA database with the Rank Product generated lists of DEGs from both this study and the *cesA4/irx5* microarray experiment [37]. For ease of computation, each probe comprised equal numbers (in this case 200) of the top up and downregulated DEGs, ranked by gene pfp (FDR) score.

**Figure 7:**
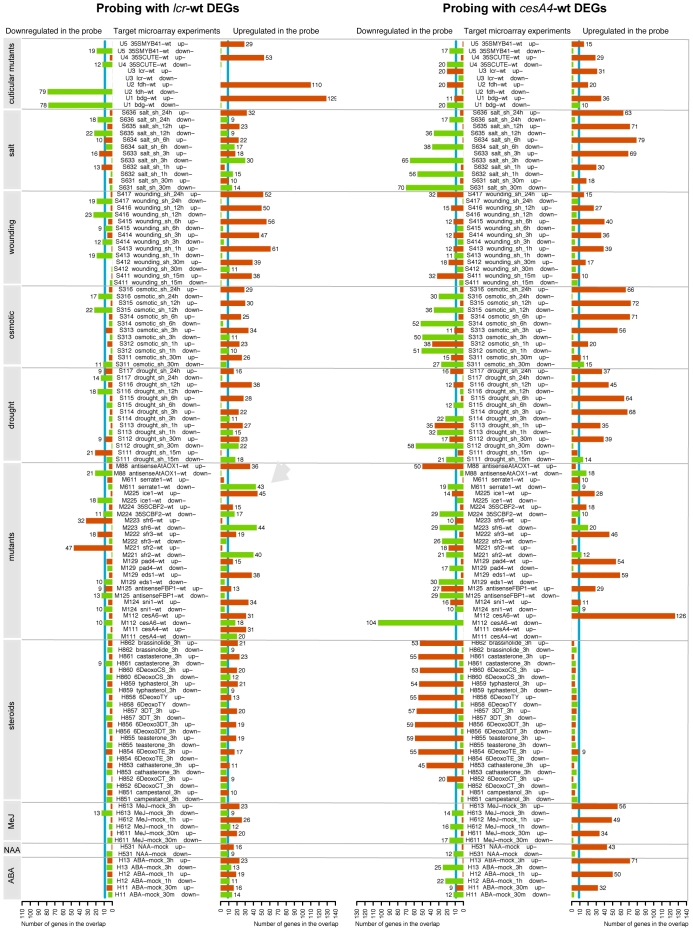
Meta-analysis of differential expressed genes (DEGs) in the cell wall and cuticle-deficient mutants. The bars illustrate gene overlaps for *lcr* and *cesA4/irx5*-differentially regulated genes when compared to a subset of DEG lists in the MASTA database (MicroArray overlap Search Tool and Analysis), which contains over 600 microarray contrasts (e.g. mutant vs. wild type, or treatment vs. control comparisons). For the best consistency, the DEGs in MASTA were selected using the Rank Product method [31]. The top 200 upregulated and downregulated genes from each contrast were used for comparisons. The number at each bar indicates the number of genes detected in the overlap between a query and a target DEG list. Arrow points to the overlap between genes upregulated in *lcr* and those downregulated in *se*. Shown are only the numbers >9 that correspond to the level of P<7.1×10 ^−05^ (vertical blue lines). The overlap values above this threshold were considered to be statistically significant.

The original MASTA output files are too long to be included in their entirety, and only selected subsets (126 out of 1208 overlaps) are, therefore, shown as examples in Figure 7 for *lcr* and *cesA4/irx5*. For each probe, a MASTA search performs pairwise testing for overlaps between up and downregulated genes in the probe and target DEG lists, thus totaling four combinations for each comparison. Following the customary terminology used in genetic analysis, in MASTA a coupling-phase overlap refers to an overlap between up and upregulated genes, or between down and downregulated genes, whereas repulsion-phase overlap refers to an overlap between up and downregulated genes, or between down and upregulated genes in the probe and target DEG lists, respectively.

The MASTA revealed that the DEGs in cuticular mutants were in the coupling phase with those in the CUTE plants [19], with 53 genes (P<2.0×10^−64^) in the overlap between the upregulated genes (Figure 7). The *cesA4/irx5* mutation induced a number of genes which are shared with cuticular mutants, but gene overlaps varied between 29 and 36 (P<5.3×10^−27^ and P<6.2×10^−37^, respectively) (Figure 7).

It is apparent from the comparative analysis that *lcr* transcriptional responses are quite similar to those induced by wounding. The overlap between upregulated genes was conspicuously like the early wounding response (15 min after wounding), but it became stronger at subsequent points in time. Although wounding-inducible genes showed statistically significant overlaps with DEGs in *cesA4/irx5*, the salt treatment, osmotic stress and drought appeared to misregulate most of the similar set of genes in *cesA4/irx5*. Large gene overlaps were noticeable for both upregulated and downregulated genes (Figure 7), with salt stress being the most similar to *cesA4/irx5*.

Remarkable differences between *lcr* and *cesA4/irx5* were seen in the overlaps with transcriptional responses to growth regulators: steroids, methyl jasmonate (MeJ), naphthaleneacetic acid (NAA) and abscisic acid (ABA). While hardly any overlaps above the threshold line were detected with the genes downregulated in *lcr*, the strong repulsion-phase overlaps were displayed with genes downregulated in *cesA4/irx5*. This suggests that a number of steroid-inducible genes (from 50 to 60) are suppressed in the *cesA4/irx5* plant, thereby evidencing the fact that steroid hormones play an essential role in the *cesA4/irx5* phenotype. Adding to the difference between cuticular and cell wall integrity signaling pathways, was the presence of strong coupling phase overlaps between *cesA4/irx5* DEGs and those induced by MeJ, NAA and ABA (Figure 7). The involvement of ABA and jasmonic acid (JA), predicted by MASTA, was in accordance with the results of Hernandez-Blanco [37] and co-workers, who used the Genevestigator Meta-Analyzer tools (www.genevestigator.ethz.ch/at/) to compare selected upregulated genes which showed fold-change values >2. Remarkably, although *cesA4/irx5* was not tested, *irx1* and *irx3* were crossed with two ABA-insensitive mutants, *abi1* and *aba3*, as well as the double homozygous mutants, *irx1 abi1* and *irx3 aba3*, and appeared to be unviable two to three weeks post-germination.

Therefore, based on these findings, we propose that in contrast to *cesA4/irx5*, major gene expression changes in *lcr* are induced independently of MeJ, NAA and ABA, and brassinosteroid signaling. Taken together, these data suggest that the underlying response mechanisms in the *cesA4/irx5* and *lcr* mutants to cell wall and cuticle defects, respectively, are sufficiently different.

### Identification of *SERRATE* among putative enhancers and suppressors by the meta-analysis of microarray data

The suppressor/enhancer screens involving the mutagenesis of the targeted mutant provide a solution to the problem of revealing additional genes in a given pathway. Alternatively, a set of known mutants may be crossed with the mutant of interest to make a series of double mutants, then enabling their phenotypes to be evaluated. The public availability of various mutant microarray datasets provides an opportunity to rapidly assess the potential of a large number of genes as genetic modifiers by using MASTA prior to the crosses. When resulting from probing with the mutant of interest, the coupling and repulsion phase overlaps indicate the presence of putative enhancers and suppressors, respectively.

To identify these, we surveyed the most significant gene overlaps with the mutant microarray datasets. A particularly noticeable case in Figure 7 was the coupling-phase overlaps of *cesA4/irx5* with *pad4* (54 upregulated genes, P<3.7×10^−66^) and *eds1* (59 upregulated genes, P<4.1×10^−75^). This suggested that both could be enhancers of *cesA4/irx5* in double mutants. *EDS1* and *PAD4* encode lipase-like proteins which can form a heterodimer and are required for the accumulation of the plant defense signal, salicylic acid [38]. *Eds1*, but not *pad4*, showed a coupling-phase overlap with *lcr* (38 upregulated genes, P<6.3×10^−40^), suggesting that it could enhance the *lcr* phenotype in double mutants, whereas *pad4* could not.

Three cases in Figure 7, which we considered to be particularly relevant as suppressors, were the recessive mutations in *SERRATE (SE), SENSITIVE TO FREEZING2 (SFR2)* and *SENSITIVE TO FREEZING6 (SFR6)*. From 23 to 45 (P<3.1×10^−19^ to 6.3×10^−51^), the genes that were repressed in the *se, sfr2* and *sfr6* mutants were induced in *lcr*, *fdh* and *bdg*, exhibiting a repulsion phase overlap.

The *se-1* mutants exhibit conspicuous leaf serration, and are affected, not only in other aspects of leaf development, but also in embryogenesis, flowering time and seedling responses to the hormone, ABA. The stronger *se* alleles, namely *se-2* and *se-3*, severely disrupt both meristem activity and leaf polarity [39]. SERRATE (At2g27100) is a zinc-finger protein which has been shown to participate in both RNA splicing and the processing of pri-miRNA transcripts into mature miRNAs [39]–[43]. The MASTA search suggests that SE has a different role in the cell wall stress pathway, with 19 downregulated genes in a coupling-phase overlap with *cesA4/irx5* (Figure 7).

We reasoned that if *SE* is required for the induction of the responses in cuticular mutants, some aspects of their phenotypes should be the opposite to those of *se* because MASTA reveals a repulsion phase overlap for their misregulated genes. We had previously noticed that *lcr* and *bdg* do, indeed, possess smooth-edged elongated leaves [14],[16], and this case has, therefore, been selected for further testing.

**Figure 8:**
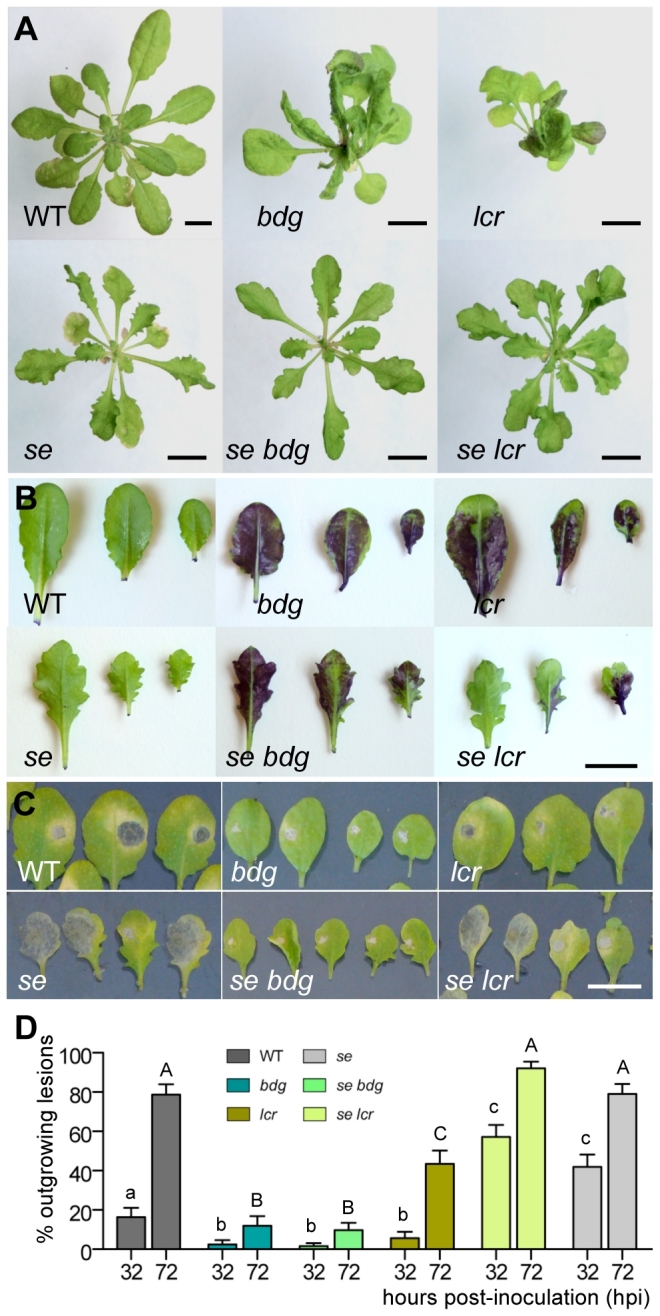
Suppression of organ fusions, TB staining, and resistance to *B. cinerea* in the *se lcr* and *se bdg* double mutants. (A) When grown under standard conditions (short-day photoperiod) in a greenhouse for four and a half weeks, *se lcr* and *se bdg* are phenotypically indistinguishable from *se* (*se* corresponds to the *se-1* allele). Bars are 1 cm in (A,B,C). (B) TB staining differentiates *se lcr* from *lcr*, *bdg* and *se bdg*. (C) Percentage of outgrowing lesions at 36 and 72 hpi (mean±se) following infection with *B. cinerea*. At least 40 leaves were used per genotype, and the statistical significance was calculated using Fisher's exact test. Letters indicate significant differences in series between genotypes as determined by pairwise comparisons (P<0.05).

From the above analysis with MASTA, one can anticipate the suppression of either phenotype in double mutants, although given the fact that SE acts in the context of a multiprotein complex which is involved in RNA processing, *se* is more likely to be epistatic to the cuticular genes. To corroborate this prediction, we obtained double mutants with the weak *se-1* allele [41]. The Figure 8A shows that both *se lcr* and *se bdg* feature serrated leaves and, essentially, look like *se* plants [41]; (*se fdh* is not yet available because the genes are tightly linked on the long arm of chromosome 2). Remarkably, under normal growth conditions, the double mutants failed to develop the ectopic organ fusions, leaf deformations and plant architecture that were characteristic of the single cuticular mutants in this class (Figure 8A) [9],[14],[16].

The TB staining phenotype of *lcr* could be significantly reverted in a *se* mutant background (Figure 8B). After infection with *B. cinerea*, *se lcr* and *se* plants tended to have larger lesion areas than wild types (Figure 8C) (the lesion areas are not shown as the lesions often covered the entire leaf surface in *se* and *se lcr*). The rate of infection was higher in *se lcr* and *se* mutants than that in wild type plants (Figure 8D). Remarkably, leaves of *se bdg* exhibited TB staining pattern and resistance to *B. cinerea* similar to that in *bdg* (Figure 8).

These findings suggest that secondary phenotypes in distinct cuticular mutants result from the induction of a response, which requires SE. This is also evidence that MASTA provides a powerful way of identifying suppressors and enhancers in the pathway of interest.

## Discussion

### The diverse phenotypes of cuticular mutants suggest the involvement of a damage response mechanism

It might be anticipated that cuticular mutants would, in general, have reduced levels of cutin monomers or wax and display conspicuous cuticle defects, however our data suggest a more complex picture when considering the cuticular mutants which display the organ fusion phenotype. We showed that the cuticle is highly disorganized in *lcr*, with cutin-like depositions and cavities in the inner layers of the cell wall. This closely resembles the cuticle of *bdg* and the CUTE plants [14],[19] as well as, to a lesser extent, *ace/hth* [27]. Yet, we also demonstrated that TEM did not reveal any visual aspects in the *fdh* cuticle which appear to be different from wild-type. However, these organ-fusion mutants are noticeably similar in the accumulation of higher levels of wax and cutin constituents in the residual bound lipids. This sets them apart from the wild-type-looking plants of cuticular mutants which exhibit a concomitant lowering of the levels of cutin components, such as *lacs2* and *att1* [21],[44],[45]. We also found that the chemical composition of the cutin, as revealed by the analysis of leaf residual bound lipids, does not show a decrease in ω-hydroxy and α,ω-dicarboxy fatty acids in *lcr*; there was also no reduction in the lengths of the fatty acid chains in *fdh*.

It is, therefore, becoming evident that a simple case scenario does not seem to be plausible for all cuticular mutants, meaning that other mechanisms need to be taken into account in order to understand the precise nature of their phenotypes and the role of cuticle in development and immunity. It may be proposed that some cuticular mutations induce a kind of a damage response that is similar to the activation of the cell wall integrity pathway in response to cell wall disrupting drugs and mutations in fungi and plants [37],[46].

Consistent with the previously observed induction of stress and defense-related genes in some *cesA* mutants [37] and the CUTE plants [23], we found by microarray gene expression profiling in *lcr*, *fdh* and *bdg* that a number of upregulated genes fall into this class. The GO analysis suggests that the functions of these genes are mainly associated with the cell wall.

We showed that misregulated genes in cuticular and cell wall mutants substantially overlap, but these genes are significantly more similar to each other than to those in the *cesA4/irx5* mutants. This may be indicative of the existence of specific signaling routes which engage transcriptional control mechanisms in the cuticular mutants. The fact that cuticular mutations confer a highly pleiotropic phenotype, including organ deformations and fusions, the extensive branching, delayed senescence and resistance to the fungal necrotroph *B. cinerea* [9],[10] that were not observed in cell wall mutants is in accord with this notion.

### Genetic dissection of the response to cuticular damage

Using biochemical analytical techniques may make it difficult or impossible to separate the overlapping loss-of-function and response phenotypes, thereby emphasizing the role of genetic approaches. To further assess the complexity of the factors responsible for the range of cuticular phenotypes, we used a meta-analytical methodology, MASTA, which has involved a reappraisal and re-analysis of several hundred Arabidopsis microarray datasets from public databases and authors [33]. MASTA is based on the proof-of-concept study showing that, a reference database of expression profiles which correspond to diverse chemical treatments and mutations in yeast can be used to functionally annotate uncharacterized genes and pharmacological perturbations in this substance [47].

Using this bioinformatics tool for the *in silico* suppressor screen, we identified *SE* as a gene which is epistatic to *lcr* and *bdg*. This prediction has been supported by three lines of evidence. First, the double mutants, *se lcr* and *se bdg*, failed to produce featured morphological changes, including organ fusions, in particular. Second, the *se lcr* mutant lost its characteristic TB staining on the epidermis surface. Third, *se lcr* lost resistance to the necrotroph, also indicating that *se* is an essential factor contributing to the complex phenotypes of cuticular mutants.

The ectopic organ fusion phenotype results from enhanced cell adhesion in epidermal cells and is interesting, because cell adhesion is a fundamental process underlying development. While adhesion between plant cells is generally established when cells are formed during cytokinesis, cells dynamically regulate adhesion, and may undergo separation or establish fusions de novo in a controlled manner, with pollen tube growth and carpel fusion being the best-known examples of the latter process [48],[49]. This suggests that there are multiple mechanisms by which plants can regulate adhesion between cells.

Organ fusions in the *fdh* mutant of Arabidopsis offered a genetic proof that the developmental program, normally limited to the gynoeceum, could be induced in the whole plant [12]. In addition to *fdh*, other Arabidopsis mutants have been reported to exhibit organ fusions and impaired morphogenesis [15], [16], [19], [27], [50]–[57]. However, the function of cuticle in these processes remained open to question. Given the role that the cuticle plays in the isolation of plant surfaces, it is necessary to study the corresponding genes in the cuticle context, particularly because most of the mutants seem to be the consequence of lesions in the genes which encode lipid-modifying enzymes. Remarkably, cell wall-related genes are prominent in the overlap which is comprised of the commonly upregulated genes in the cuticular mutants and downregulated genes in *se* (Table 1 and Table S3), Further experiments would be required to determine which of these genes, or others as yet not recognized, are involved in epidermal adhesion and leaf morphogenesis. Since the latest version of the Arabidopsis genome annotation (TAIR8) contains information about 27235 protein-coding genes, and the ATH1 array represents approximately 23750 genes (87%) [58], we note that about 13% of the downstream genes of interest may escape identification in both our and Lobbes' microarray experiments [43]. The discovery of epistatic effects could be the first step towards identifying the cell-surface molecules and understanding genetics of the putative interactional mechanisms which underlie the organ fusion phenotype.

The mechanism by which the distorted cuticle leads to a strong resistance to *B. cinerea* is also unclear but may involve an inhibitory action of plant-derived toxins such as camalexin and a higher permeability of cuticle to these compounds or fungal elicitors [59],[60]. Fast induction of camalexin biosynthesis genes in wounded and infected plants accounts for the strong immunity against *B. cinerea* [59]. MASTA revealed that DEGs in *se* (data not shown) were in the repulsion phase with those in cuticular mutants and wounded plants, suggesting that wounding response may be compromised by the *se* mutation in *se lcr*. However, we did not find that *se bdg* plants were more susceptible (and become less stainable) than *bdg*, indicating a greater complexity of the antifungal resistance in cuticular mutants.

The function of the nuclear-localized SE protein in the regulation of the pleiotropic phenotypes of cuticular mutants also remains to be determined. So far it is known that, like DICER-LIKE1 (DCL1) and HYPONASTIC LEAVES1 (HYL1), SE is required for miRNA biogenesis but not for sense post-transcriptional gene silencing [42],[43]. In *se*, 20 upregulated genes have been identified as known targets of miRNAs and/or transacting siRNAs (ta-siRNAs) [43]. However, only downregulated genes in *se* show an overlap with DEGs in the cuticular mutants, and none of the *se* downregulated genes present on the ATH1 chip are known miRNA targets [43]. Interactions between the SE, DCL1 and HYL1 proteins are essential for the efficient and precise processing of pri-miRNA [61], although MASTA does not predict the presence of epistatic interactions with *dcl1* and *hyl1* (data not shown). The possibility that SE may be associated with an alternative pathway in RNA signaling warrants further investigation. Nevertheless, it is an important finding that *se* is epistatic to the mutations in the distinct epidermis-specific cuticular genes *LCR* and *BDG*, suggesting that the impaired cuticle triggers specific cell signaling pathway. Accordingly, this study offers an intriguing and unexpected insight into how cuticle formation, cell adhesion and morphogenesis in plants may be co-regulated.

## Materials and Methods

### Plant material and growth conditions

All plants used in this study were derived from *Arabidopsis thaliana* (L.) ecotype Columbia (Col-O).

The mutant alleles used were: *fdh-3940S1* [20], *lcr-3P77* [16], *bdg-2* [14], *se-1* [41], *lacs2-3* [21] and *pad3-1* [25]. Arabidopsis plants were grown in a greenhouse or controlled environment chamber at 22 to 23°C and 50 to 60% humidity under a short day photo-period (8-h light) for the first 6–7 weeks, and then under a long day photo-period (16-h light) if not, otherwise, indicated.

### Generation of transgenic plants and GFP expression studies

The putative 1.3-kb promoter region of the *LCR* gene was amplified by PCR with the P*LCR*-H (TGAACTCCAAAGCTTTACATGACTACATCG) and P*LCR*-Xb (CTCTAGATCTCCTCATAAACTTGGAGTGA) primers (*Hind*III and *Xba*I sites are underlined in the primer sequences), and cloned as a HindIII/XbaI fragment into the pBgreen binary vector [17]. Wild-type *Arabidopsis thaliana* Col-0 plants were transformed with the resulting pB*LCR*:GFP by vacuum-infiltration [62], and BASTA-resistant transgenic plants were selected. The analysis of GFP expression with a confocal laser scanning microscope (Leica TCS 4D) was performed in tissue sections from the pB*LCR*:GFP plants as described [14].

### *In situ* hybridization

*In situ* hybridization was performed using the same antisense digoxigenin-labeled riboprobes that were derived from a cDNA clone of the *DAISY* gene, and an epidermis-specific control, as in Franke [34]. The hybridization products were revealed by an immunohistochemical reaction after incubation with an alkaline phosphatase-conjugated anti-digoxigenin antibody. Probe preparation, the hybridization procedure, and immunohistochemical detection were conducted as previously described [17].

### Transmission electron microscopy (TEM)

To study the fine structure of the cuticle, plants were grown for 4–5 weeks under long day conditions. Tissue samples were embedded, and ultra-thin sections (50–70 nm) were contrasted as described in [21] (*fdh*) and [19] (wild type and *lcr*). The samples were examined with a Phillips CM12 transmission electron microscope or a Philips CM 100 BIOTWIN electron microscope. The details of the procedures can be found in our previous work [19],[21].

### Scanning electron microscopy (SEM)

To examine wax coating, rosette leaves from 6–7 week-old plants grown under short day conditions, as well as stem internodes (4th and 5th) from 12 week-old plants (8 weeks under short and 4 weeks under long-day conditions) were prepared for cryo-SEM. The samples were deep-frozen and sputtered with palladium using the K1250X cryogenic preparation system (Emitech, England). Leaf surfaces were examined with a Zeiss SEM SUPRA 40VP microscope.

### Characterization of cuticle permeability

Toluidine blue staining was performed according to Tanaka and co-workers [26]. Chlorophyll leakage from rosette leaves into ethanol was performed according to Lolle and co-workers [13], with modifications as previously described [14].

### *Botrytis cinerea* growth, inoculation, and disease progression measurements

*B. cinerea* strains 2100 and B05.10 were grown on the potato dextrose agar (Difco Laboratories) at 22°C for seven to nine days. Spores were harvested and washed twice in water, and filtered through Miracloth (Calbiochem). Inoculations were made as previously described [24]. Briefly, rosette leaves of four-week-old soil-grown plants were placed in square Petri dishes containing 0.8% agar with petioles in the medium. Four µl of a suspension containing 5×10^5^ conidiospores/mL in 1/4-strength potato dextrose broth (Difco Laboratories) were deposited on the detached leaves. A single drop was applied to each leaf between the middle vein and the edge of the leaf, and the leaves were incubated under continous light at 22–24°C. Disease symptoms were scored at 3 days after challenge. High humidity was maintained by sealing the dishes with Parafilm. Macroscopic images were acquired with a digital camera (Sony DSC-W120) and the lesion area was measured in pixels using Image J (software available at http://rsbweb.nih.gov/ij/) and then converted to square millimeters. The inoculation experiments were repeated three times with detached leaves using at least 50 leaves per genotype and once *in planta* with similar results.

### Analysis of cuticular lipid polyesters and wax

Fatty acid composition analyses of residual bound lipids and wax were performed as previously described in detail [14],[27]. Cutin and wax constituents were separated and identified by GC–MS using a gas chromatograph 6890N equipped with a quadrupole mass selective detector 5973N (Agilent Technologies, Boeblingen, Germany). The composition analysis in the *lcr* and *fdh* leaves was performed twice. In each experiment, plants were grown for 10–11 weeks under short day conditions prior to tissue harvest. For wax analysis, plants were grown for seven weeks under the same conditions.

### Hybridization to Affymetrix ATH1 chips and quality control

Mutants (*bdg*, *lcr*, *fdh*) and WT (Col-0) were grown side by side in a growth chamber under short day conditions at 20°C/18°C. Plants were five and a half weeks old when tissue was harvested. Three independent samples, each containing typical young leaves (from 2 mm to 1 cm long) from 15 plants, were prepared per plant type. Total RNA was extracted using the RNeasy Plant Mini Kit (Qiagen) according to the manufacturer's instructions. RNA concentration and quality were assessed with agarose gel electrophoresis. The samples were sent to the Integrated Functional Genomics (IFG) platform of the Westfalian-Wilhelms-University (Muenster, Germany; http://campus.uni-muenster.de/ifg.html) for a further quality check, preparation of biotin-labeled cRNA probes, hybridization to GeneChip Arabidopsis ATH1 Genome Arrays and scanning of the slides. All of the above steps were performed according to the manufacturer's instructions. For each plant type, we had three biological and no technical replicates.

The pre-processing of raw data and the Affymetrix MAS5.0 Quality Control tests were performed using Bioconductor packages (http://www.bioconductor.org) and custom written scripts in the R programming environment (http://www.r-project.org). For the quality control tests, we used the SimpleAffy package [63]. The quality-control measures indicated that the 12 microarrays used in this study show no systematic signal distortion, similar scale factors, and adequate background levels, sufficient percentage of genes called “present” and acceptable performance in 3/5 ratio tests (Figure S5). The microarray data reported in this paper have been deposited in the Gene Expression Omnibus (GEO) database, www.ncbi.nlm.nih.gov/geo (accession no. GSE15105).

### Defining the lists of misregulated genes

Statistical analysis was performed using custom written scripts for the Bioconductor RankProd package [64]. To estimate the false discovery rate (FDR), pfp (false positive predictions) values have been calculated from 100 permutations. The predicted differentially expressed genes (DEGs) have been ordered by increasing pfp value. For this report, a 5% (0.05) pfp cutoff has been applied to the definition of the DEGs in the mutants.

### Meta-analysis of microarrays and in silico suppressor/enhancer screens

The meta-analytic software, MASTA (MicroArray overlap Search Tool and Analysis), was written to run in R (http://www.r-project.org).

A database for MASTA was created. To this end, Affymetrix raw data files (CEL files) were downloaded from the Gene Expression Omnibus (GEO, http://www.ncbi.nlm.nih.gov/geo), ArrayExpress (http://www.ebi.ac.uk/microarray-as/ae/), TAIR AtGenExpress (http://www.arabidopsis.org/index.jsp), the Integrated Microarray Database System (http://ausubellab.mgh.harvard.edu/imds), or via the NASC Affywatch subscription service (http://nasc.nott.ac.uk/). Some CEL files have been obtained from authors' websites or from the authors directly. At the time of this study, the MASTA database comprised DEGs for over 600 contrasts (mutant vs. wild type or treatment vs. control comparisons) calculated by using custom RankProd scripts in the R programming environment. Other portions of MASTA included the overlap analysis and plotting routines (details will be published elsewhere). The RankProd-selected DEGs were ordered by increasing pfp value, and the top 200 DEGs from the lists containing up and downregulated genes were used for the overlap analysis in this report. Output PDF files from MASTA were imported to Adobe Illustrator (Adobe Systems, San Jose, CA) for assembly. The statistical significance of the overlap between two DEG lists was determined by using the online program available at http://elegans.uky.edu/MA/progs/overlap_stats.html.

MASTA will be made available for viewing and downloading at http://bar.utoronto.ca/ (The Bio-Array Resource for Arabidopsis Functional Genomics).

### Confirmation of microarray results by semi-quantitative RT–PCR

In brief, 0.5 µg aliquots of the total RNA of each hybridization sample, treated with DNase I, were reverse-transcribed to the first-strand cDNA with a One-Step RT–PCR Kit (Qiagen). These cDNAs were used as templates for PCR under the following conditions: denaturation at 94°C (1 min); N_opt_ cycles of 94°C (1 min), 58°C (45 sec) and 72°C (30 sec, except for At4g30280 where it was 1 min); then 72°C final extension (15 min). Gene-specific primer pairs are listed in Table S4. The expression of *ACTIN2* (At3g18780) was analyzed as an internal control. The semi-quantitative RT–PCR reactions were optimized for number of cycles N_opt_ to ensure product intensity within the linear phase of amplification (close to the the lower limit of the linear range) for each gene (Table S4). PCR fragments were quantified (Typhoon 8600 PhosphorImager, Amersham Biosciences) following electrophoresis in ethidium bromide-containing agarose gels.

### Genotyping of the *serrate* cross-progeny

The segregating F2 populations (about 200 plants each) derived from the *lcr*×*se-1* and *bdg*×*se-1* crosses respectively were tested in a blind two-stage screen for the presence of mutant and wild-type alleles in the *LCR*, *BDG* and *SERRATE* loci by PCR. The following PCR conditions were used: denaturation at 94°C (2 min); 36 cycles of 94°C (30 sec), 58°C (30 sec) and 72°C (50 sec); then final extension at 72°C (5 min).

At the first stage, DNA was isolated in 96-well blocks, based on the method described previously [65]. To genotype the *LCR* locus, amplification products were loaded on a 1.5% agarose gel. For the *BDG* and *SE* loci, half of the PCR products were first digested with *Mwo*I or *Bfu*CI (NEB) respectively, and then the undigested and digested PCR products were loaded onto a high-resolution agarose gel (3% GenAgarose Tiny HT, Genaxxon Bioscience).

At the second stage, candidate double mutants were re-screened by repeating DNA isolation with the DNeasy Plant Mini Kit (Qiagen) and PCR analysis. Several plants have been identified for each double mutant type. The allele-specific primers and PCR products are listed in Table S5; the sequence of the *lcr-3P77* transposon insertion allele was deposited in the GenBank under accession number FJ767868.

## Supporting Information

Figure S1 Ultrastructure of ectopic organ-fusion zones in *lcr* and *fdh*. TEM images illustrate the zones of contact between the epidermal cells of different organs. (A,B) Two rosette leaves (lf) in *lcr* form a fusion which is intervened by irregular depositions of electron-dense materials (A), or without traces of the cuticle proper interposed between the organs (B). (C) Under mechanical stress, organs fail to complete fusions but remain strung together. Shown are two leaves in the *lcr* mutant. (D,E) Fusions between two rosette leaves (D) and a petiole (po) and a leaf (E) in *fdh*. Arrowheads indicate fusion zones. (F–H) The cuticle covering the floral organs has a smooth surface on the adaxial side of wild-type sepals (se) (F) and a corrugated wavy form on the abaxial side (G). The anther (an) cuticles showed varied degrees of undulation (H). (I,J,K) Fusions in *fdh* between the petal (pe) and the adaxial side of sepal (I), two anther portions (J) and two sepals (K). The fusion in I is limited to the selective adhesion sites (arrowheads). The undulation amplitude decreases in the fusion zone (arrowhead) (J). The waving pattern, characteristic of the abaxial sepal cuticle, disappeared following the fusion in K. Bars are 200 nm.
(0.47 MB PDF)

Figure S2 Supplemental analysis of leaf residual bound lipids. The increase in C18:2 α,ω-diacids in *lcr* and *fdh* was also observed in two independent experiments. Values are mean±standard error for six replicates, each containing leaves from at least 15 plants. Stars indicate a significant Mann-Whitney test (two-tailed, P<0.05) for mutant versus wild-type.
(0.07 MB PDF)

Figure S3 Wax deposition on adaxial rosette leaf surface in three cuticular mutants and wild-type plants. At least four rosette leaves per plant type were examined under SEM. Note the smooth surface of the wild-type and epicuticular wax crystalloids on the leaf surface in the mutants. Bar = 5 µm.
(0.45 MB PDF)

Figure S4 SEM micrographs of stem surfaces. Two stems (4th and 5th internodes) of 12-week-old plants (first eight weeks short day, then four weeks long day) were examined per plant type. Wild-type waxes mainly contain dendrites (1), rodlets (2) and umbrellas (3), whereas mutant samples generally display horizontal plates (4). More rounded crystals could also be observed in all three mutants, although they are especially conspicuous in *bdg*. Bars are 5 µm (upper panel) and 1 µm (lower panel).
(7.27 MB PDF)

Figure S5 Microarray Quality Control (QC) measures. The plot from the package, SimpleAffy [63], which shows the QC measures recommended by Affymetrix. All ATH1 chips passed the tests, including wt2 and bdg1, for which the GAPDH3′GAPDH5′ ratios are slightly disturbed. For a detailed explanation, refer to the SimpleAffy manual (http://bioconductor.wustl.edu/BioC2.1/bioc/html/simpleaffy.html) and to the ‘Expression analysis fundamentals’ manual, which is available on the Affymetrix website (http://www.affymetrix.com/).
(0.23 MB PDF)

Table S1 Upregulated genes in three cuticular mutants.
(0.58 MB XLS)

Table S2 Downregulated genes in three cuticular mutants.
(0.54 MB XLS)

Table S3 Common, statistically significant DEGs in three cuticular mutants.
(0.04 MB XLS)

Table S4 Primers for semi-quantitative RT-PCR.
(0.07 MB DOC)

Table S5 Primers for genotyping.
(0.04 MB DOC)

Text S1 Survey of common, statistically significant DEGs in three cuticular mutants.
(0.21 MB PDF)

## References

[1] Jeffree CE, Kerstiens G (1996). Structure and ontogeny of plant cuticles.. Plant Cuticles: An integrated functional approach.

[2] Jeffree CE, Riederer M, Müller C (2006). The fine structure of the plant cuticle.. Biology of the Plant Cuticle.

[3] Markham KR, Ryan KG, Gould KS, Rickards GK (2000). Cell wall sited flavonoids in lisianthus flower petals.. Phytochemistry.

[4] Pollard M, Beisson F, Li Y, Ohlrogge JB (2008). Building lipid barriers: biosynthesis of cutin and suberin.. Trends Plant Sci.

[5] Nawrath C (2006). Unraveling the complex network of cuticular structure and function.. Curr Opin Plant Biol.

[6] Kunst L, Samuels AL (2003). Biosynthesis and secretion of plant cuticular wax.. Prog Lipid Res.

[7] Samuels L, Kunst L, Jetter R (2008). Sealing plant surfaces: cuticular wax formation by epidermal cells.. Annu Rev Plant Biol.

[8] Heredia A (2003). Biophysical and biochemical characteristics of cutin, a plant barrier biopolymer.. Biochim Biophys Acta-Gen Subj.

[9] Yephremov A, Schreiber L (2005). The dark side of the cell wall: Molecular genetics of plant cuticle.. Plant Biosyst.

[10] Reina-Pinto JJ, Yephremov A (2009). Surface lipids and plant defenses.. Plant Physiol Biochem.

[11] Tanaka H, Machida Y, Riederer M, Müller C (2006). The cuticle and cellular interactions.. Biology of the Plant Cuticle.

[12] Lolle SJ, Cheung AY, Sussex IM (1992). *Fiddlehead* - an Arabidopsis mutant constitutively expressing an organ fusion program that involves interactions between epidermal-cells.. Dev Biol.

[13] Lolle SJ, Berlyn GP, Engstrom EM, Krolikowski KA, Reiter WD (1997). Developmental regulation of cell interactions in the Arabidopsis *fiddlehead-1* mutant: A role for the epidermal cell wall and cuticle.. Dev Biol.

[14] Kurdyukov S, Faust A, Nawrath C, Bar S, Voisin D (2006). The epidermis-specific extracellular BODYGUARD controls cuticle development and morphogenesis in Arabidopsis.. Plant Cell.

[15] Lolle SJ, Hsu W, Pruitt RE (1998). Genetic analysis of organ fusion in *Arabidopsis thaliana*.. Genetics.

[16] Wellesen K, Durst F, Pinot F, Benveniste I, Nettesheim K (2001). Functional analysis of the *LACERATA* gene of Arabidopsis provides evidence for different roles of fatty acid -hydroxylation in development.. Proc Natl Acad Sci U S A.

[17] Efremova N, Schreiber L, Bar S, Heidmann I, Huijser P (2004). Functional conservation and maintenance of expression pattern of *FIDDLEHEAD*-like genes in Arabidopsis and Antirrhinum.. Plant Mol Biol.

[18] Suh MC, Samuels AL, Jetter R, Kunst L, Pollard M (2005). Cuticular lipid composition, surface structure, and gene expression in Arabidopsis stem epidermis.. Plant Physiol.

[19] Sieber P, Schorderet M, Ryser U, Buchala A, Kolattukudy P (2000). Transgenic Arabidopsis plants expressing a fungal cutinase show alterations in the structure and properties of the cuticle and postgenital organ fusions.. Plant Cell.

[20] Yephremov A, Wisman E, Huijser P, Huijser C, Wellesen K (1999). Characterization of the *FIDDLEHEAD* gene of Arabidopsis reveals a link between adhesion response and cell differentiation in the epidermis.. Plant Cell.

[21] Bessire M, Chassot C, Jacquat AC, Humphry M, Borel S (2007). A permeable cuticle in Arabidopsis leads to a strong resistance to *Botrytis cinerea*.. EMBO J.

[22] Tang D, Simonich MT, Innes RW (2007). Mutations in LACS2, a long-chain acyl-coenzyme A synthetase, enhance susceptibility to avirulent *Pseudomonas syringae* but confer resistance to *Botrytis cinerea* in Arabidopsis.. Plant Physiol.

[23] Chassot C, Nawrath C, Metraux JP (2007). Cuticular defects lead to full immunity to a major plant pathogen.. Plant J.

[24] Ferrari S, Plotnikova JM, De Lorenzo G, Ausubel FM (2003). Arabidopsis local resistance to *Botrytis cinerea* involves salicylic acid and camalexin and requires EDS4 and PAD2, but not SID2, EDS5 or PAD4.. Plant J.

[25] Glazebrook J, Ausubel FM (1994). Isolation of phytoalexin-deficient mutants of *Arabidopsis thaliana* and characterization of their interactions with bacterial pathogens.. Proc Natl Acad Sci U S A.

[26] Tanaka T, Tanaka H, Machida C, Watanabe M, Machida Y (2004). A new method for rapid visualization of defects in leaf cuticle reveals five intrinsic patterns of surface defects in Arabidopsis.. Plant J.

[27] Kurdyukov S, Faust A, Trenkamp S, Bar S, Franke R (2006). Genetic and biochemical evidence for involvement of HOTHEAD in the biosynthesis of long-chain alpha-,omega-dicarboxylic fatty acids and formation of extracellular matrix.. Planta.

[28] Pruitt RE, Vielle-Calzada JP, Ploense SE, Grossniklaus U, Lolle SJ (2000). *FIDDLEHEAD*, a gene required to suppress epidermal cell interactions in Arabidopsis, encodes a putative lipid biosynthetic enzyme.. Proc Natl Acad Sci U S A.

[29] Franke R, Briesen I, Wojciechowski T, Faust A, Yephremov A (2005). Apoplastic polyesters in Arabidopsis surface tissues - A typical suberin and a particular cutin.. Phytochemistry.

[30] Bonaventure G, Beisson F, Ohlrogge J, Pollard M (2004). Analysis of the aliphatic monomer composition of polyesters associated with Arabidopsis epidermis: occurrence of octadeca-cis-6, cis-9-diene-1,18-dioate as the major component.. Plant J.

[31] Breitling R, Armengaud P, Amtmann A, Herzyk P (2004). Rank products: a simple, yet powerful, new method to detect differentially regulated genes in replicated microarray experiments.. FEBS Lett.

[32] Breitling R, Herzyk P (2005). Rank-based methods as a non-parametric alternative of the T-statistic for the analysis of biological microarray data.. J Bioinform Comput Biol.

[33] Reina-Pinto JJ, Voisin D, Teodor R, Yephremov A (2009). Probing differentially expressed genes against a microarray database for *in silico* suppressor/enhancer and inhibitor/activator screens.. Plant J (in press).

[34] Franke R, Höfer R, Briesen I, Emsermann M, Efremova N (2008). The *DAISY* gene from Arabidopsis encodes a fatty acid elongase condensing enzyme involved in the biosynthesis of aliphatic suberin in roots and the chalaza-micropyle region of seeds.. Plant J.

[35] Provart NJ, Zhu T, Spang R, Beziat P, Vingron M (2003). A browser-based Functional Classification SuperViewer for Arabidopsis genomics.. Currents in Computational Molecular Biology.

[36] MAQC Consortium (2006). The MicroArray Quality Control (MAQC) project shows inter-and intraplatform reproducibility of gene expression measurements.. Nat Biotechnol.

[37] Hernandez-Blanco C, Feng DX, Hu J, Sanchez-Vallet A, Deslandes L (2007). Impairment of cellulose synthases required for Arabidopsis secondary cell wall formation enhances disease resistance.. Plant Cell.

[38] Feys BJ, Moisan LJ, Newman MA, Parker JE (2001). Direct interaction between the Arabidopsis disease resistance signaling proteins, EDS1 and PAD4.. EMBO J.

[39] Grigg SP, Canales C, Hay A, Tsiantis M (2005). SERRATE coordinates shoot meristem function and leaf axial patterning in Arabidopsis.. Nature.

[40] Laubinger S, Sachsenberg T, Zeller G, Busch W, Lohmann JU (2008). Dual roles of the nuclear cap-binding complex and SERRATE in pre-mRNA splicing and microRNA processing in *Arabidopsis thaliana*.. Proc Natl Acad Sci U S A.

[41] Prigge MJ, Wagner DR (2001). The Arabidopsis *SERRATE* gene encodes a zinc-finger protein required for normal shoot development.. Plant Cell.

[42] Yang L, Liu Z, Lu F, Dong A, Huang H (2006). SERRATE is a novel nuclear regulator in primary microRNA processing in Arabidopsis.. Plant J.

[43] Lobbes D, Rallapalli G, Schmidt DD, Martin C, Clarke J (2006). SERRATE: a new player on the plant microRNA scene.. EMBO Rep.

[44] Xiao FM, Goodwin SM, Xiao YM, Sun ZY, Baker D (2004). Arabidopsis CYP86A2 represses *Pseudomonas syringae* type III genes and is required for cuticle development.. EMBO J.

[45] Schnurr J, Shockey J, Browse J (2004). The acyl-CoA synthetase encoded by *LACS2* is essential for normal cuticle development in Arabidopsis.. Plant Cell.

[46] Humphrey TV, Bonetta DT, Goring DR (2007). Sentinels at the wall: cell wall receptors and sensors.. New Phytol.

[47] Hughes TR, Marton MJ, Jones AR, Roberts CJ, Stoughton R (2000). Functional discovery via a compendium of expression profiles.. Cell.

[48] Verbeke JA (1992). Fusion events during floral morphogenesis.. Annu Rev Plant Biol.

[49] Mollet J-C, Faugeron C, Morvan H, Roberts JA, Gonzalez–Carranza Z (2007). Cell adhesion, separation and guidance in compatible plant reproduction.. Plant Cell Separation and Adhesion: Wiley-Blackwell.

[50] Tanaka H, Watanabe M, Sasabe M, Hiroe T, Tanaka T (2007). Novel receptor-like kinase ALE2 controls shoot development by specifying epidermis in Arabidopsis.. Development.

[51] Bird D, Beisson F, Brigham A, Shin J, Greer S (2007). Characterization of Arabidopsis ABCG11/WBC11, an ATP binding cassette (ABC) transporter that is required for cuticular lipid secretion.. Plant J.

[52] Panikashvili D, Savaldi-Godstein S, Mandel T, Yifhar T, Franke RB (2007). The Arabidopsis *DESPERADO/AtWBC11* transporter is required for cutin and wax secretion.. Plant Physiol.

[53] Ukitsu H, Kuromori T, Toyooka K, Goto Y, Matsuoka K (2007). Cytological and biochemical analysis of *COF1*, an Arabidopsis mutant of an ABC transporter gene.. Plant Cell Physiol.

[54] Krolikowski KA, Victor JL, Wagler TN, Lolle SJ, Pruitt RE (2003). Isolation andcharacterization of the Arabidopsis organ fusion gene *HOTHEAD*.. Plant J.

[55] Baud S, Bellec Y, Miquel M, Bellini C, Caboche M (2004). *gurke* and *pasticcino3* mutants affected in embryo development are impaired in acetyl-CoA carboxylase.. EMBO Rep.

[56] Bellec Y, Harrar Y, Butaeye C, Darnet S, Bellini C (2002). PASTICCINO2 is a protein tyrosine phosphatase-like involved in cell proliferation and differentiation in *Arabidopsis*.. Plant J.

[57] Tanaka H, Onouchi H, Kondo M, Hara-Nishimura I, Nishimura M (2001). A subtilisin-like serine protease is required for epidermal surface formation in Arabidopsis embryos and juvenile plants.. Development.

[58] Redman JC, Haas BJ, Tanimoto G, Town CD (2004). Development and evaluation of an Arabidopsis whole genome Affymetrix probe array.. Plant J.

[59] Chassot C, Buchala A, Schoonbeek H, Metraux JP, Lamotte O (2008). Wounding of Arabidopsis leaves causes a powerful but transient protection against Botrytis infection.. Plant J.

[60] Chassot C, Nawrath C, Métraux JP (2008). The cuticle: Not only a barrier for plant defence.. Plant Signal Behav.

[61] Fang Y, Spector DL (2007). Identification of nuclear dicing bodies containing proteins for microRNA biogenesis in living Arabidopsis plants.. Curr Biol.

[62] Bechtold N, Ellis J, Pelletier G (1993). In-planta Agrobacterium-mediated gene-transfer by infiltration of adult *Arabidopsis thaliana* plants.. CR Acad Sci Paris, Life Sci.

[63] Wilson CL, Miller CJ (2005). Simpleaffy: a BioConductor package for Affymetrix Quality Control and data analysis.. Bioinformatics.

[64] Hong F, Breitling R, McEntee CW, Wittner BS, Nemhauser JL (2006). RankProd: a bioconductor package for detecting differentially expressed genes in meta-analysis.. Bioinformatics.

[65] Berendzen K, Searle I, Ravenscroft D, Koncz C, Batschauer A (2005). A rapid and versatile combined DNA/RNA extraction protocol and its application to the analysis of a novel DNA marker set polymorphic between *Arabidopsis thaliana* ecotypes Col-0 and Landsberg *erecta*.. Plant Methods.

