# Supplementary material for: Dissection of the Complex Phenotype in Cuticular Mutants of Arabidopsis Reveals a Role of SERRATE as a Mediator
Source: PLoS Genet. 2009 Oct 30;5(10):e1000703. doi: 10.1371/journal.pgen.1000703 (PMC2760142; doi:10.1371/journal.pgen.1000703)
Supplement: Figure S1 — Ultrastructure of ectopic organ-fusion zones in lcr and fdh. TEM images illustrate the zones of contact between the epidermal cells of different organs. (A,B) Two rosette leaves (lf) in lcr form a fusion which is intervened by irregular depositions of electron-dense materials (A), or without traces of the cuticle proper interposed between the organs (B). (C) Under mechanical stress, organs fail to complete fusions but remain strung together. Shown are two leaves in the lcr mutant. (D,E) Fusions between two rosette leaves (D) and a petiole (po) and a leaf (E) in fdh. Arrowheads indicate fusion zones. (F–H) The cuticle covering the floral organs has a smooth surface on the adaxial side of wild-type sepals (se) (F) and a corrugated wavy form on the abaxial side (G). The anther (an) cuticles showed varied degrees of undulation (H). (I,J,K) Fusions in fdh between the petal (pe) and the adaxial side of sepal (I), two anther portions (J) and two sepals (K). The fusion in I is limited to the selective adhesion sites (arrowheads). The undulation amplitude decreases in the fusion zone (arrowhead) (J). The waving pattern, characteristic of the abaxial sepal cuticle, disappeared following the fusion in K. Bars are 200 nm. (0.47 MB PDF) [file pgen.1000703.s001.pdf]

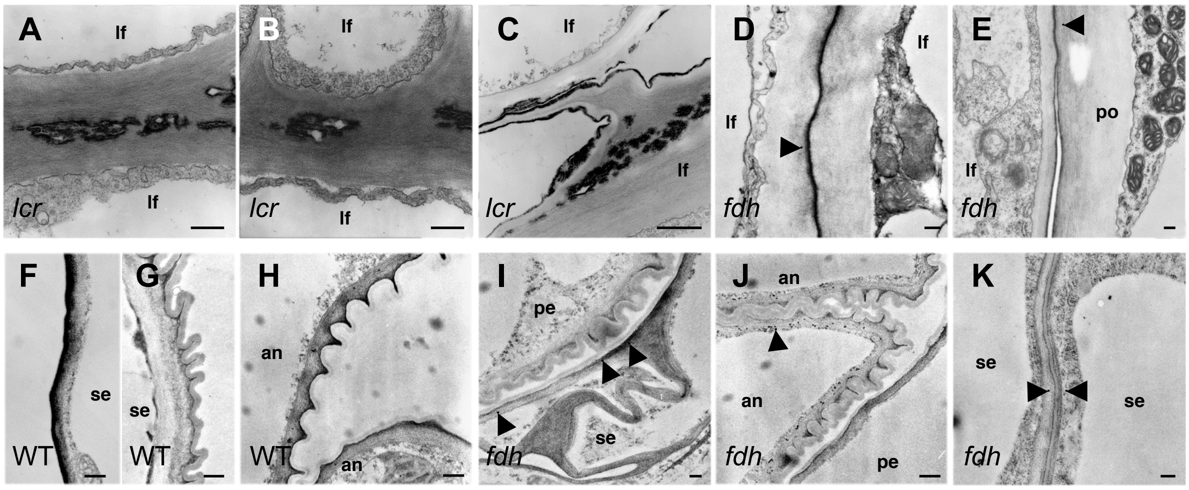

Figure S1. Ultrastructure of ectopic organ-fusion zones in *lcr* and *fdh*.

TEM images illustrate the zones of contact between the epidermal cells of different organs.

(A, B) Two rosette leaves (lf) in *lcr* form a fusion which is intervened by irregular depositions of electron-dense materials (A), or without traces of the cuticle proper interposed between the organs (B).
(C) Under mechanical stress, organs fail to complete fusions but remain strung together. Shown are two leaves in the *lcr* mutant.
(D, E) Fusions between two rosette leaves (D) and a petiole (po) and a leaf (E) in *fdh*. Arrowheads indicate fusion zones.
(F-H) The cuticle covering the floral organs has a smooth surface on the adaxial side of wild-type sepals (se) (F) and a corrugated wavy form on the abaxial side (G). The anther (an) cuticles showed varied degrees of undulation (H). The cuticle in petals is similar to that in anthers with regard to undulation (data not shown).
(I, J, K) Fusions in *fdh* between the petal (pe) and the adaxial side of sepal (I), two anther portions (J) and two sepals (K). The fusion in I is limited to the selective adhesion sites (arrowheads). The undulation amplitude decreases in the fusion zone (arrowhead) (J). The waving pattern, characteristic of the abaxial sepal cuticle, disappeared following the fusion in K.
Bars are 200 nm.
