# Supplementary material for: Dissection of the Complex Phenotype in Cuticular Mutants of Arabidopsis Reveals a Role of SERRATE as a Mediator
Source: PLoS Genet. 2009 Oct 30;5(10):e1000703. doi: 10.1371/journal.pgen.1000703 (PMC2760142; doi:10.1371/journal.pgen.1000703)
Supplement: Figure S2 — Supplemental analysis of leaf residual bound lipids. The increase in C18:2 α,ω-diacids in lcr and fdh was also observed in two independent experiments. Values are mean±standard error for six replicates, each containing leaves from at least 15 plants. Stars indicate a significant Mann-Whitney test (two-tailed, P<0.05) for mutant versus wild-type. (0.07 MB PDF) [file pgen.1000703.s002.pdf]

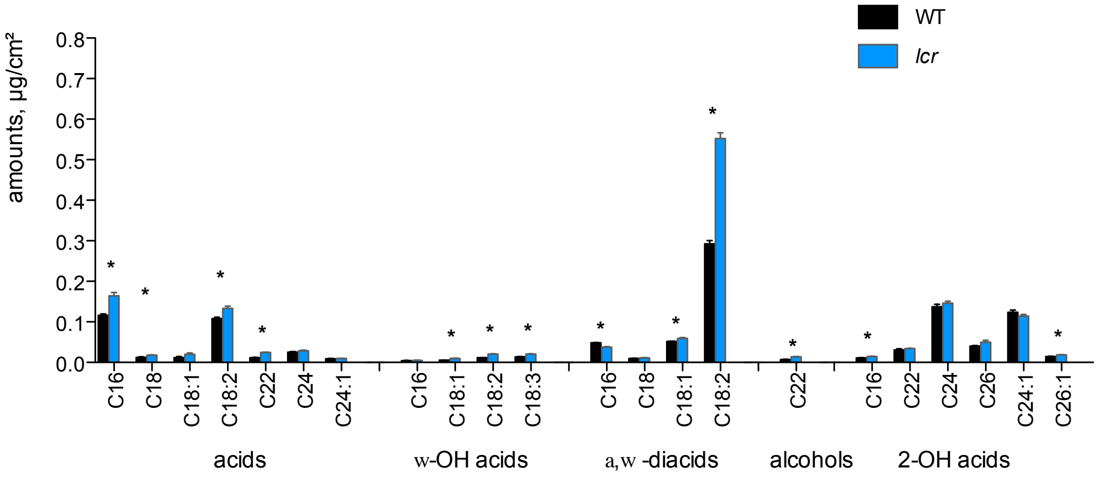

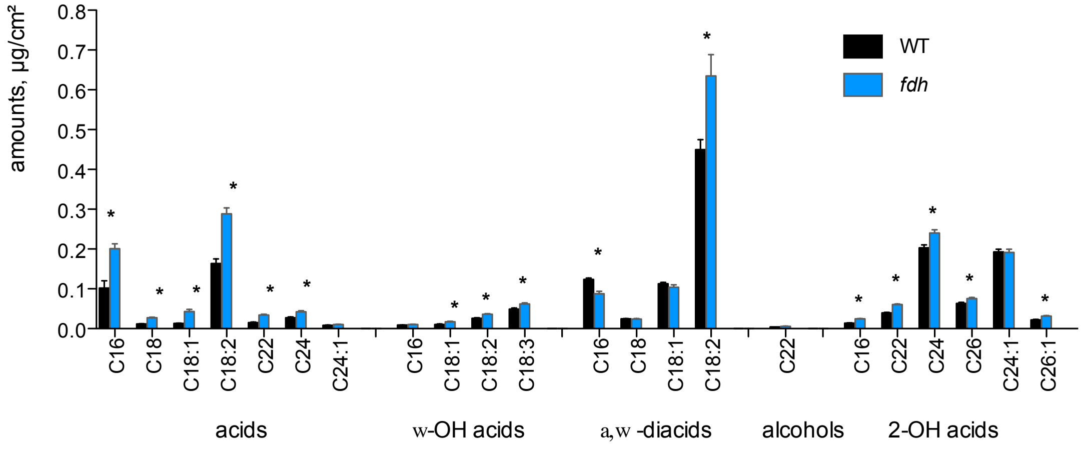

Figure S2. Supplemental analysis of residual bound lipids.
The increase in C18:2 α,ω-diacids in *lcr* and *fdh* was also observed in two independent experiments. Values are means ± standard errors for six replicates each containing leaves from at least ten plants. Stars indicate a significant Mann-Whitney test (two-tailed, $P < .05$) for mutant versus wild type.
