# Supplementary material for: Dissection of the Complex Phenotype in Cuticular Mutants of Arabidopsis Reveals a Role of SERRATE as a Mediator
Source: PLoS Genet. 2009 Oct 30;5(10):e1000703. doi: 10.1371/journal.pgen.1000703 (PMC2760142; doi:10.1371/journal.pgen.1000703)
Supplement: Figure S3 — Wax deposition on adaxial rosette leaf surface in three cuticular mutants and wild-type plants. At least four rosette leaves per plant type were examined under SEM. Note the smooth surface of the wild-type and epicuticular wax crystalloids on the leaf surface in the mutants. Bar = 5 µm. (0.45 MB PDF) [file pgen.1000703.s003.pdf]

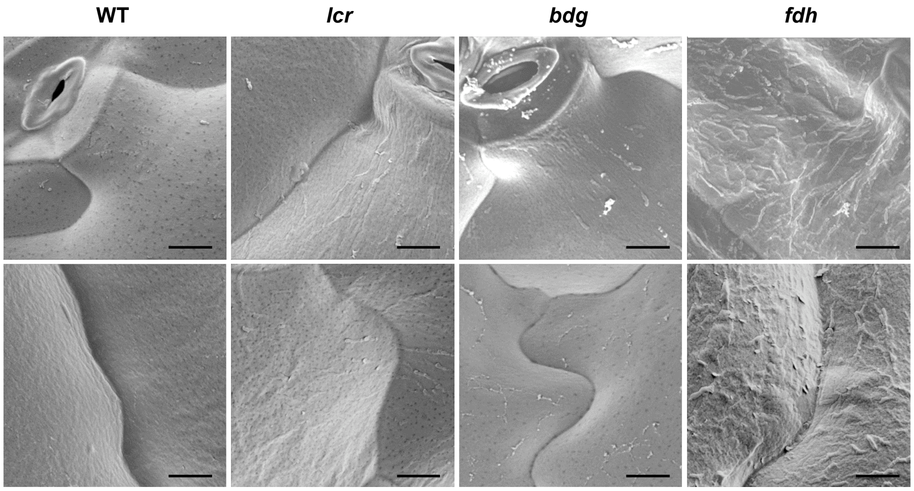

Figure S3. Wax deposition on adaxial rosette leaf surface in three cuticular mutants and wild type plants.

At least four rosette leaves per plant type were examined under SEM. Note the smooth surface of the wild type and epicuticular wax crystalloids on the leaf surface in the mutants.
Bars are 5 µm.
