# Supplementary material for: Dissection of the Complex Phenotype in Cuticular Mutants of Arabidopsis Reveals a Role of SERRATE as a Mediator
Source: PLoS Genet. 2009 Oct 30;5(10):e1000703. doi: 10.1371/journal.pgen.1000703 (PMC2760142; doi:10.1371/journal.pgen.1000703)
Supplement: Figure S4 — SEM micrographs of stem surfaces. Two stems (4th and 5th internodes) of 12-week-old plants (first eight weeks short day, then four weeks long day) were examined per plant type. Wild-type waxes mainly contain dendrites (1), rodlets (2) and umbrellas (3), whereas mutant samples generally display horizontal plates (4). More rounded crystals could also be observed in all three mutants, although they are especially conspicuous in bdg. Bars are 5 µm (upper panel) and 1 µm (lower panel). (7.27 MB PDF) [file pgen.1000703.s004.pdf]

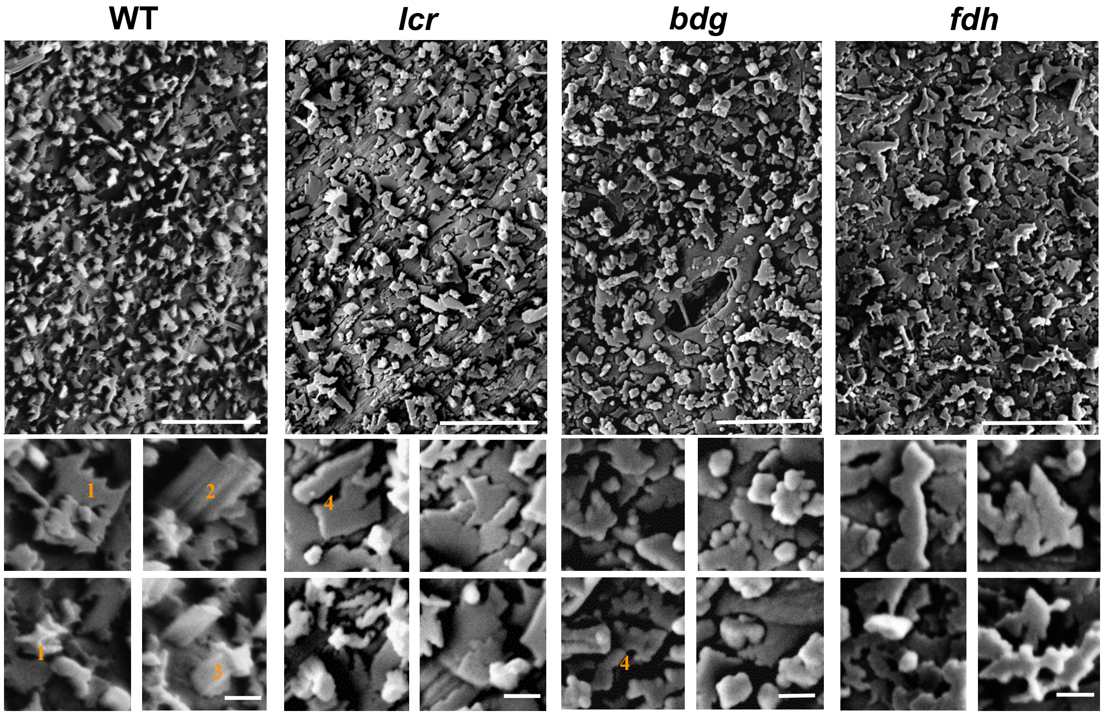

Figure S4. SEM micrographs of stem surfaces.

Two stems (4th and 5th internodes) of 12-weeks-old plants (first eight weeks short day then four weeks long day) were examined per plant type. Wild-type waxes mostly contain dendrites (1), rodlets (2) and umbrellas (3)) whereas mutant samples mainly display horizontal plates (4). Bars are 5 μm (upper panel) and 1 μm (lower panel).
