# Supplementary material for: Dissection of the Complex Phenotype in Cuticular Mutants of Arabidopsis Reveals a Role of SERRATE as a Mediator
Source: PLoS Genet. 2009 Oct 30;5(10):e1000703. doi: 10.1371/journal.pgen.1000703 (PMC2760142; doi:10.1371/journal.pgen.1000703)
Supplement: Figure S5 — Microarray Quality Control (QC) measures. The plot from the package, SimpleAffy [63], which shows the QC measures recommended by Affymetrix. All ATH1 chips passed the tests, including wt2 and bdg1, for which the GAPDH3′GAPDH5′ ratios are slightly disturbed. For a detailed explanation, refer to the SimpleAffy manual (http://bioconductor.wustl.edu/BioC2.1/bioc/html/simpleaffy.html) and to the ‘Expression analysis fundamentals’ manual, which is available on the Affymetrix website (http://www.affymetrix.com/). (0.23 MB PDF) [file pgen.1000703.s005.pdf]

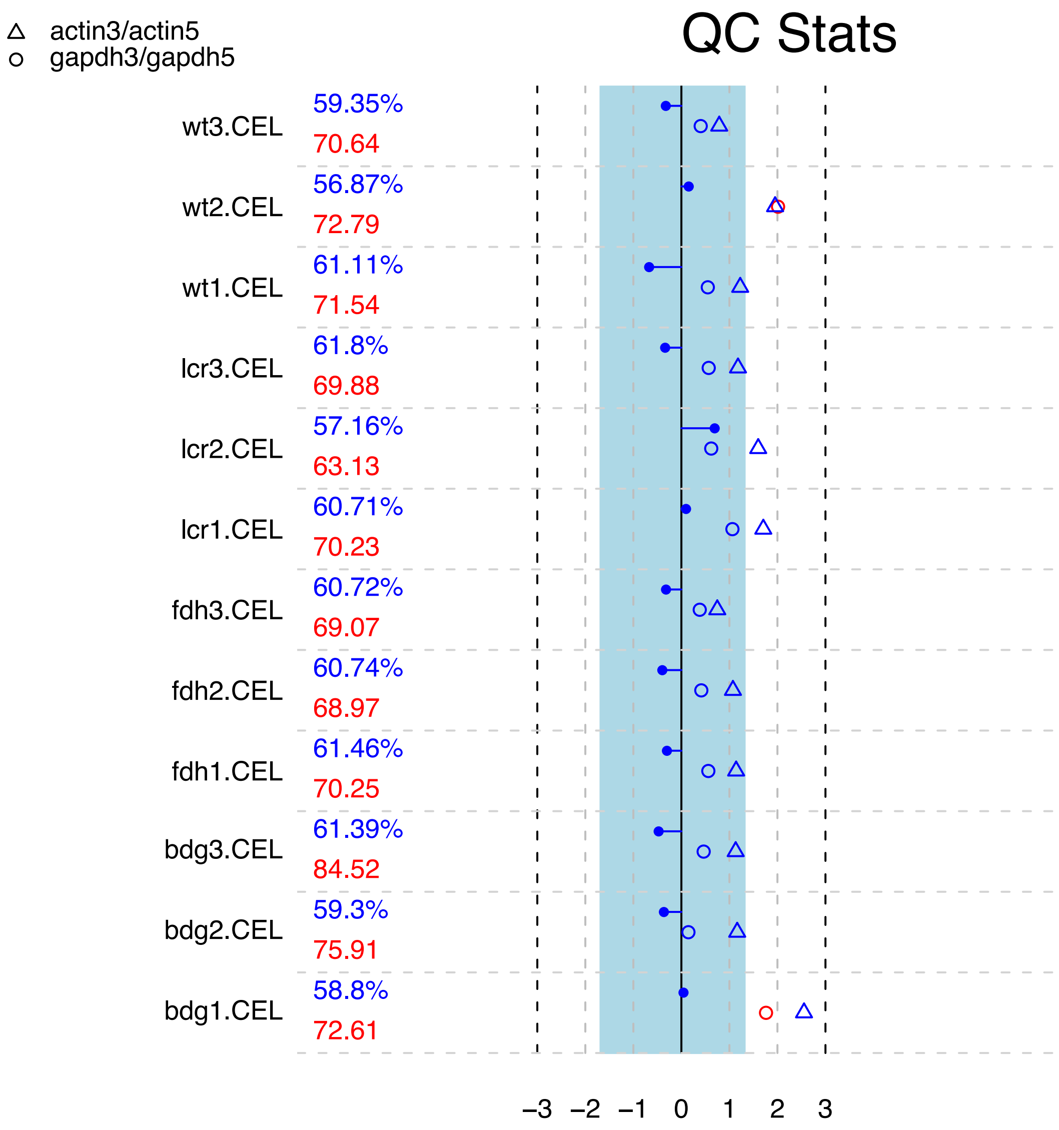

Figure S5. Microarray Quality Control (QC) measures.
The plot from the package SimpleAffy (Miller, 2005) showing Quality Control (QC) measures recommended by Affymetrix. All ATH1 chips passed the tests, including two chips, wt2 and bdg1, for which GAPDH3'GAPDH5' ratios are slightly disturbed.
For detailed explanation, refer to the SimpleAffy manual (http://bioconductor.wustl.edu/BioC2.1/bioc/html/simpleaffy.html) and to the 'Expression analysis fundamentals' manual, available on Affymetrix website (http://www.affymetrix.com/).
