# Supplementary material for: Dissection of the Complex Phenotype in Cuticular Mutants of Arabidopsis Reveals a Role of SERRATE as a Mediator
Source: PLoS Genet. 2009 Oct 30;5(10):e1000703. doi: 10.1371/journal.pgen.1000703 (PMC2760142; doi:10.1371/journal.pgen.1000703)
Supplement: Table S4 — Primers for semi-quantitative RT-PCR. (0.07 MB DOC) [file pgen.1000703.s009.doc]

**Table S4**. Primers for semi-quantitative RT-PCR.

| gene | locus ID | primer | primer sequence (5'-->3') | N_opt_ ^*^ |
| --- | --- | --- | --- | --- |
| *LTP3* | At5G59320 | D173 | GCA GGT AGC TTG GCT CCA TGT GCA AC | 24 |
|  |  | D174 | TAT TTT ATT CTA GTA CTT CTG GTA A |  |
| *LTP4* | At5G59310 | D175 | GTG GCA CAG TGG CAA GTA GCT TGA G | 24 |
|  |  | D176 | GAT AGC CGT CTT ATT TTA CGT ATA CG |  |
| *LTP* | At5G01870 | D169 | GTG TTA CCA CTA TGC CTG CTT CTT GCT | 30 |
|  |  | D170 | CAC TAA ACT CCG GTT ACA TTA GAC GA |  |
| *LTP2* | At2G38530 | D171 | GGA GTG ATG AAG TTG GCA TGC ATG | 26 |
|  |  | D172 | ATT GAA AAG AGT ACA GCC ATT CG |  |
| *PPT1* | At5G47330 | D185 | AAG GTT TGA AGC GGT CTG GTG TTG C | 28 |
|  |  | D186 | TCC CTT GAT TAG CTC ATC TGC TAA C |  |
| *RAP2.6* | At1G43160 | D189 | TGA TTA CCG GTT CAG CTG TGA CTA A | 38 |
|  |  | D190 | CAA AGC GTT GAC AAT ATG TTA GTT A |  |
| *XTH18* | At4G30280 | D187 | AAC AAT ACA TTT GAG ATA TCA ATA CA | 36 |
|  |  | D195 | TAC ACA AAC ACC GCA TAC ATA TGA G |  |
| *DAISY* | At1G04220 | D177 | TCT CTT CGC CGC TCT TCT TAT CTT | 26 |
|  |  | D178 | AGC GGT CGG AGG AGC GGT TAG |  |
| *CER4* | At4G33790 | D179 | ACT CAC GTG CTT CCT CTG TGA TCT TG | 24 |
|  |  | D180 | GTC GTC CCA ATC GAG AAC CTT TGG AT |  |
| *WAX2* | At5G57800 | D021 | AAG CAT CCT GAC CTT AGA GTT CGT GTG GTT CAT | 24 |
|  |  | D022 | TAA GAC CAT ACT TCA TGG CTG CTT CCC ACA |  |
| *HTL7* | At5g51950 | D193 | ATG CAG ACA AGC CGT ACT ACT AGT | 30 |
|  |  | D194 | ATA CTC TTT AAG TTA ATC ATA CAT T |  |
| *RAP2.6L* | At5g13330 | D191 | ACC AGA CCA AGA TCA ACC AAG A | 38 |
|  |  | D192 | TTA TTC TCT TGG GTA GTT ATA A |  |
| *CER1* | At1G02205 | D027 | CAG GAA CGG AGA GGT GTA TAT CCA CAA CCA T | 22 |
|  |  | D028 | CTA TCA ATG CTG GTG TGG TAT GAT AGA TAC |  |
| *ACTIN2* | At3g18780 | D019 | AGA GAT TCA GAT GCC CAG AAG TCT TGT TCC | 22 |
|  |  | D020 | AAC GAT TCC TGG ACC TGC CTC ATC ATA CTC |  |

Note: Gene specific primers for *RAP2.6*, *DAISY*, and *ACTIN2* were from ^1^, ^2^ .

* Number of cycles has been optimized for each gene and allowed detection of the products in the linear range of the amplification

1. Che, P., Lall, S., Nettleton, D. & Howell, S.H. Gene expression programs during shoot, Root, and callus development in Arabidopsis tissue culture. *Plant Physiol.* **141**, 620-637 (2006).

2. Franke, R. et al. The *DAISY* gene from Arabidopsis encodes a fatty acid elongase condensing enzyme involved in the biosynthesis of aliphatic suberin in roots and the chalaza-micropyle region of seeds. *Plant J* **57**, 80-95 (2008).
