# Supplementary material for: Dissection of the Complex Phenotype in Cuticular Mutants of Arabidopsis Reveals a Role of SERRATE as a Mediator
Source: PLoS Genet. 2009 Oct 30;5(10):e1000703. doi: 10.1371/journal.pgen.1000703 (PMC2760142; doi:10.1371/journal.pgen.1000703)
Supplement: Table S5 — Primers for genotyping. (0.04 MB DOC) [file pgen.1000703.s010.doc]

**Table S5**. Primers for genotyping.

Primers

| locus | primer name | orientation | primer sequence, 5'-3' |
| --- | --- | --- | --- |
| *LCR* | D235 | forward | CAT GCA ATT AAT AGA CAC GAG AAT A |
| *LCR* | D236 | reverse | CAA CGA GAT ATG AAT GTG AGC CAT A |
| *LCR* | D240 | reverse | GAG CGT CGG TCC CCA CAC TTC TAT AC |
| *BDG* | D233 | forward | AAT CGA CAT CAC ATG TTC CTT AGT A |
| *BDG* | D234 | reverse | TCG ACC ACC GTG GAG TTG ACA GTG A |
| *SE* | D237 | forward | CCC ACC ACC AAT GCT GAT GCC TGT T |
| *SE* | D238 | reverse | CAA GCT CCT GTA ATC AAT AAC |

Primer combinations and products

| allele | combination in PCR | PCR product, bp | digestion products, bp | enzyme |
| --- | --- | --- | --- | --- |
| *LCR* | D235/D236 | 838 |  |  |
| *lcr-3P77* | D235/240 | 555 |  |  |
| *BDG* | D233/D234 | 740 | 283;274;110;73 | *Mwo*I |
| *bdg-2* | D233/D234 | 733 | 550;110;73 | *Mwo*I |
| *SE* | D237/D238 | 453 | 172;110;69;60;42 | *Bfu*CI |
| *se-1* | D237/D238 | 446 | 225;110;69;42 | *Bfu*CI |
