# Supplementary material for: Dissection of the Complex Phenotype in Cuticular Mutants of Arabidopsis Reveals a Role of SERRATE as a Mediator
Source: PLoS Genet. 2009 Oct 30;5(10):e1000703. doi: 10.1371/journal.pgen.1000703 (PMC2760142; doi:10.1371/journal.pgen.1000703)
Supplement: Text S1 — Survey of common, statistically significant DEGs in three cuticular mutants. (0.21 MB PDF) [file pgen.1000703.s011.pdf]

Text S1. Survey of common, statistically significant DEGs in three cuticular mutants

Cell wall genes

Candidates in the top GO class include three glycine rich proteins (GRPs), At4g21620, At2g05540 and At3g20470 (GPR5), a proline-rich protein (PRP), At2g16630, a hydroxyproline rich protein (HRGP), At5g09530, and one of the extensins, At1g76930 (EXT4), which belong to a distinct type of HRGPs (Table 1). All of these represent structural cell wall proteins which could confer additional properties on cell walls, such as mechanical strength [1]. For example, extensins are known to be responsible for cell wall rigidification. They can undergo insolubilization in the wall matrix through the peroxidase-mediated formation of intra and intermolecular cross-links [2].

GRP5 is specifically expressed in the epidermis of aerial organs, and may be necessary in the protoderm to strengthen the epidermal cell wall, thus delimiting growth [3]. However, the functions of this protein and other GRPs remain unclear (see [4] and [5] for reviews).

ARABINOGALACTAN-PROTEIN2 (AGP2; At2g22470) (Table 1) also belongs to the HRGP family. AGPs are typically heavily modified by O-glycosylation, with polysaccharides constituting up to 95% of the mass of the molecule. AGPs and extensins are, in fact, the most well represented structural proteins in the cell wall [6].

The other group in this GO class is represented by the xyloglucan endotransglycosylases/hydrolases (XTHs) At4g30280 (*XTH18*), At4g30290 (*XTH19*) and At4g25810 (*XTH23*). XTHs are extracellular enzymes which act to remodel xyloglucan cross-links in the cell wall [6, 7]. Xyloglucan comprises over 20% of the primary cell wall carbohydrates in Arabidopsis, and XTHs are thought to have significant roles in maintaining the cellulose–xyloglucan network [7].

The genes which have been shown or suggested to be involved in the biosynthesis of cuticular lipids are not particularly annotated with a GO term yet, and they are not, therefore, reported by the Classification SuperViewer. However, we did notice the presence of this class of genes among the commonly misregulated genes in Table 1.

In fact, eight lipid transfer protein (*LTP*) genes are shortlisted in this Table, and four of them, At5g59320 (*LTP*3), At5g59310 (*LTP*4), At5g01870, and At2g38530 (*LTP*2), were ranked as the highest by the Rank Product method (Table 1). Most of these LTPs belong to the type 1 and type 5 groups [8] and seem to be upregulated in the growing epidermis [9]. Proteins from this large mutltigenic family are likely to be involved in lipid transportation: in vitro assays show that they are able to bind fatty acids and transfer phospholipids between membranes [10]. The direct involvement of these (and other) LTPs in the transport of wax or cutin precursors has, however, yet to be demonstrated.

Also highly upregulated in the cuticular mutants was the ABCG1 (At2g39350) half-transporter (Table 1). Two closely related proteins, CER5/WBC12/ABCG12 (hereafter ABCG12) and WBC11/DSO/PEL1/COF1/ABCG11) (hereafter

ABCG11), have previously been shown to be required for wax secretion, and could probably form functional lipid transporters through homo and heterodimerization [11]. In addition to the *eceriferum* phenotype, ABCG11 (At1g17840) seems to be required for the secretion of cutin monomers, since its mutants and antisense transgenic plants accumulate less cutin (35% in *wbc11-3* compared to wild type) and exhibit an organ fusion phenotype that is not observed in *wbc12* [12]. The impact of closely related *AtWBC1/ABCG1* on the cuticle is not known from the Arabidopsis mutants. However, NtWBC1 was found to be specifically expressed in the secretory cells of stigmas, as well as in anthers in tobacco, and could be involved in the transport of lipid to the exudates [13].

The upregulation of *DAISY* and *ECERIFERUM4 (CER4)* (Table 1), which are the two genes associated with the biosynthesis, or modification, of VLCFAs, suggests that these are important processes which contribute to cuticle homeostasis. *CER4* (At4g33790), which is an epidermis-specific gene [14], encodes an alcohol-forming fatty acyl-CoA reductase that is responsible for the production of primary alcohols during wax biosynthesis. *DAISY*, which has been mentioned earlier in this paper, is induced in the mutant epidermis where it could potentially boost the fatty acid elongation required for wax biosynthesis. *DAISY* is related to *FDH* and two epidermis-specific KCSs, CUT1 and KCS1, which are involved in cuticular wax biosynthesis [15-18]. Since *DAISY* is also expressed in the inner layers of the developing leaves, it is likely to have a role in the biosynthesis of other lipids as well.

CER3/WAX2/YRE/FLP1/PEL6 (At5g57800) and *CER1* (At1g02205) encode two putative related, epidermis-specific, enzymes [19,20]. Both genes are indispensable for the normal biosynthesis of wax, although the exact molecular functions of the corresponding proteins have yet to be determined. While *CER3/WAX2/YRE/FLP1/PEL6* was selected for Table 1, *CER1* was not because of some inconsistency in the expression values for two ATH1 gene-chip probes (264146_at and 264147_at) which correspond to *CER1*. However, if the expression values for these probes were combined rather than calculated separately, it is possible to consider *CER1* as a candidate which reveals a 3-5-fold increase in the mutants over wild type (data not shown).

Another gene for which expression in the mutants is noticeably higher is *HOTHEAD-LIKE7* (At5g51950; *HTL7*) [21] (Table 1). It is homologous to the epidermis specific *ACE/HTH* gene, which encodes for a fatty acid ω-alcohol dehydrogenase which probably catalyzes the oxidation of ω-hydroxylated fatty acids to aldehydes. Cutin from the *ace/hth* mutant contains more ω-hydroxy-fatty acids and fewer α,ω-diacids [22]. At5g51950/*HTL7* is predicted by microarray analysis to be epidermis specific [9]. Therefore, one can anticipate that it directly contributes to the increase of C18:2 α,ω-diacid levels in the cuticular mutants.

The precise physiological functions of most GDSL lipases have yet to be elucidated, but it seems that they are capable of facilitating the hydrolysis of a variety of bonds, including ester, thioester and amide, in a wide range of substrates, and may occasionally act as acyltransferases [23]. One epidermis specific GDSL lipase gene from *Agave americana* L. has been characterized in detail, however its role in the biosynthesis of epidermal lipids is poorly understood [23]. Our microarray analysis has identified the At2g04570 gene coding for a

putative lipase of the GDSL family (Table 1). Remarkably, the same gene has been detected overexpressing the *WIN1/SHN1* gene at higher levels in *WIN1*-HA plants [24]. These plants overaccumulate cuticular waxes in leaves and stems [25, 26] and cutin [24].

The transcription factor WIN1/SHN1 belongs to subfamily V of the ERF (ethylene response factors) family [27]. Four transcription factors, At1g64380 from subfamily I, At1g21910 from subfamily II, RAP2.6 (At1g43160) and RAP2.6L (At5g13330) from subfamily X were identified by our analysis. At1g64380 appears to be a good candidate for being an activating factor involved in wax biosynthesis, since the overexpression of two *Medicago truncatula* transcription factor genes, WXP1 and WXP2, also led to increased leaf wax accumulation [28]. Both genes belong to the same ERF subfamily,(I), as does At1g64380.

Defense genes

This category includes genes that are known to be involved in various cellular functions. For example, *RESPONSE TO DESICCATION26* (At4g27410; *RD26*) reached high levels in the three cuticular mutants. This transcription factor belongs to the NAC plant specific transcription factor family [29], and is induced in response to dehydration, osmotic stress and abscisic acid [30]. Based on the comparison of microarray-expression data, the ERF transcription factor RAP2.6, which is referred to above, has been grouped by clustering it with four transcription factor genes that are activated by many types of pathogens [31]. One of these genes is *WRKY6* (At1g62300), which is also strongly upregulated in the cuticular mutants (Table 1).

To activate plant defenses, transcription factors target a diversity of downstream genes which are related to particular functions, such as anti-oxidative action.

Two aldo/keto reductase genes, At2g37760 and At2g37770, were found to be upregulated in the mutants. In general, aldo/keto reductases are NADPH-dependent oxidoreductases, and through their activity they contribute to the detoxification process by reducing oxidation products. Interestingly, it has been reported that At2g37770 encodes a putative D-glucuronic acid reductase which is involved in the biosynthesis of vitamin C (L-ascorbic acid) [32]. Vitamin C has multiple functions in the cell, including acting as a radical scavenger and an enzyme co-factor. It is the most abundant water soluble antioxidant in plant cells, and is found in most subcellular compartments, including the cell wall [32]. The transcripts of the raffinose synthase *ARABIDOPSIS THALIANA SEED IMBIBITION2* (*ATSIP*-2; At3g57520) gene, which catalyses the production of raffinose family oligosaccharides (RFOs), was also expressed at higher levels in the mutant tissues. RFOs are water soluble, non reducing sugars that are used as carbon sources and antioxidants against various abiotic stresses, such as drought, cold, or salt [33].

Transcripts of *LATE EMBRYOGENESIS ABUNDANT* (*LEA*; At1g52690) are dramatically induced in the three mutants (Table 1). LEAs are highly hydrophilic protective proteins which are associated with the response to desiccation and cold shock in plants and animals. They probably act as molecular chaperones by preventing proteins from aggregating during cell stress [34].

Also related to the group of anti-stress proteins is the BLUE-COPPER-BINDING PROTEIN (BCB; At5g20230). Over-expression of BCB enhances tolerance to aluminum stress, increases

the accumulation of lignin, and reduces the levels of lipid peroxides [35]. *BCB* is rapidly upregulated after infection with non-host and avirulent *P. syringae* strains. It has been proposed that it contributes to cell wall fortifications through lignification at the inoculation sites [36].

Reactive oxygen species (ROS), such as hydrogen peroxide (H_2O_2), also play a role in signaling the activation of defense responses under biotic and abiotic stresses. At5g64120, which is upregulated in the cuticular mutants (Table 1), was characterized as encoding a cell wall bound peroxidase which is involved in ROS production and hypo-osmotic signaling [37].

Recently, it has been proposed that hexoses function as signals in the cell wall integrity pathway in plants [38]. The model hypothesizes that they are directly involved in the cell wall stress signaling by way of a sensory mechanism that perceives changes in turgor pressure and/or shearing between the plasma membrane and the cell wall [38]. The *SUGAR TRANSPORT PROTEIN13 (STP13)* (At5g26340) is one of the highly upregulated genes in mutant tissues, and is likely to be involved in this signaling process. The expression of this transmembrane transporter [39, 40] may be specific to the epidermis, as determined by the microarray analysis [9].

Several other genes which may, potentially, be involved in the signaling include *MILDEW RESISTANCE LOCUS12* (*MLO12*; At2g39200), coding for a putative receptor protein [41], and four putative protein kinases (At1g51800, At3g46280, At5g11410, At4g11890).

Increases in transcript abundance were also found in genes that are putatively involved in anti-pathogenic processes. The At2g43510 gene codes for the small Cys-rich extracellular ARABIDOPSIS THALIANA TRYPSIN INHIBITOR PROTEIN 1 (ATTL1). This defensin-like gene has been identified as a general oxidative stress marker [42]. It may be involved in pathogen defense as well as in a chitinase (At2g43620), *STRICTOSIDINE SYNTHASE3* (*SS3*; At1g74000), *ELICITOR-ACTIVATED GENE3* (*ELI3*; At4g37990) and the already mentioned *MLO12,* all of which were also upregulated in the cuticular mutants.

At5g47330, which was one of the genes with the strongest response, encodes a putative palmitoyl-protein thioesterase (PPT) which could be involved in post-translational membrane attachment and the dynamic fatty acylation of diverse proteins, including ligands, receptors and protein kinases [43].

According to our selection criteria, only two genes were found to be commonly downregulated in the three mutants. Both, *FE SUPEROXIDE DISMUTASE 1* (*FSD1*; At4g25100) and a putative glutaredoxin (At2g47880), may be involved in the regulation of the redox potential in the cell in response to stress.

References

1. Kim LJ, Brian JJ, Carolyn JS, Antony B (2003) Non-enzymic cell wall (glyco) proteins. In: Rose JKC, editor. The Plant Cell Wall. Oxford: Blackwell Publishing Ltd. pp. 111-154.
2. Humphrey TV, Bonetta DT, Goring DR (2007) Sentinels at the wall: cell wall receptors and sensors. New Phytol. 176: 7-21.
3. Sachetto-Martins G, Fernandes LD, Felix DB, de Oliveira DE (1995) Preferential transcriptional activity of a glycine-rich protein gene from *Arabidopsis thaliana* in protoderm-derived cells. Int. J. Plant Sci. 156: 460-470.
4. Sachetto-Martins G, Franco LO, de Oliveira DE (2000) Plant glycine-rich proteins: a family or just proteins with a common

motif? Biochim. Biophys. Acta-Gene Struct. Expression 1492: 1-14.

5. Ringli C, Keller B, Ryser U (2001) Glycine-rich proteins as structural components of plant cell walls. Cell. Mol. Life Sci. 58: 1430-1441.
6. Liepman AH, Cavalier DM, Lerouxel O, Keegstra K (2007) Cell wall structure, biosynthesis and assembly. In: Roberts JA, Gonzalez–Carranza Z, editors. Plant Cell Separation and Adhesion: Wiley-Blackwell. pp. 8-39.
7. Rose JKC, Braam J, Fry SC, Nishitani K (2002) The XTH family of enzymes involved in xyloglucan endotransglucosylation and endohydrolysis: current perspectives and a new unifying nomenclature. Plant Cell Physiol. 43: 1421-1435.
8. Beisson F, Koo AJK, Ruuska S, Schwender J, Pollard M, et al. (2003) Arabidopsis genes involved in acyl lipid metabolism. A 2003 census of the candidates, a study of the distribution of expressed sequence tags in organs, and a web-based database. Plant Physiol. 132: 681-697.
9. Suh MC, Samuels AL, Jetter R, Kunst L, Pollard M, et al. (2005) Cuticular lipid composition, surface structure, and gene expression in Arabidopsis stem epidermis. Plant Physiol. 139: 1649-1665.
10. Douliez JP, Michon T, Elmorjani K, Marion D (2000) Structure, biological and technological functions of lipid transfer proteins and indolines, the major lipid binding proteins from cereal kernels. J. Cereal Sci. 32: 1-20.
11. Bird DA (2008) The role of ABC transporters in cuticular lipid secretion. Plant Sci. 174: 563-569.
12. Bird D, Beisson F, Brigham A, Shin J, Greer S, et al. (2007) Characterization of Arabidopsis ABCG11/WBC11, an ATP binding cassette (ABC) transporter that is required for cuticular lipid secretion. Plant J. 52: 485-498.
13. Otsu CT, daSilva I, de Molfetta JB, da Silva LR, de Almeida-Engler J, et al. (2004) NtWBC1, an ABC transporter gene specifically expressed in tobacco reproductive organs. J. Exp. Bot. 55: 1643-1654.
14. Rowland O, Zheng H, Hepworth SR, Lam P, Jetter R, et al. (2006) *CER4* encodes an alcohol-forming fatty acyl-Coenzyme A reductase involved in cuticular wax production in Arabidopsis. Plant Physiol. 142: 866-877.
15. Todd J, Post-Beittenmiller D, Jaworski JG (1999) *KCS1* encodes a fatty acid elongase 3-ketoacyl-CoA synthase affecting wax biosynthesis in *Arabidopsis thaliana*. Plant J. 17: 119-130.
16. Fiebig A, Mayfield JA, Miley NL, Chau S, Fischer RL, et al. (2000) Alterations in *CER6*, a gene identical to *CUT1*, differentially affect long-chain lipid content on the surface of pollen and stems. Plant Cell 12: 2001-2008.
17. Millar AA, Clemens S, Zachgo S, Giblin EM, Taylor DC, et al. (1999) *CUT1*, an Arabidopsis gene required for cuticular wax biosynthesis and pollen fertility, encodes a very-long-chain fatty acid condensing enzyme. Plant Cell 11: 825-838.
18. Franke R, Höfer R, Briesen I, Emsermann M, Efremova N, et al. (2008) The *DAISY* gene from Arabidopsis encodes a fatty acid elongase condensing enzyme involved in the biosynthesis of aliphatic suberin in roots and the chalaza-micropyle region of seeds. Plant J. 57: 80-95.
19. Chen XB, Goodwin SM, Boroff VL, Liu XL, Jenks MA (2003) Cloning and characterization of the *WAX2* gene of Arabidopsis involved in cuticle membrane and wax production. Plant Cell 15: 1170-1185.
20. Aarts MGM, Keijzer CJ, Stiekema WJ, Pereira A (1995) Molecular characterization of the *CER1* gene of Arabidopsis involved in epicuticular wax biosynthesis and pollen fertility. Plant Cell 7: 2115-2127.
21. Krolikowski KA, Victor JL, Wagler TN, Lolle SJ, Pruitt RE (2003) Isolation and characterization of the Arabidopsis organ fusion gene *HOTHEAD*. Plant J. 35: 501-511.
22. Kurdyukov S, Faust A, Trenkamp S, Bar S, Franke R, et al. (2006) Genetic and biochemical evidence for involvement of HOTHEAD in the biosynthesis of long-chain alpha-,omega-dicarboxylic fatty acids and formation of extracellular matrix. Planta 224: 315-329.
23. Reina JJ, Guerrero C, Heredia A (2007) Isolation, characterization, and localization of *AgaSGNH* cDNA: a new SGNH-motif plant hydrolase specific to *Agave americana* L. leaf epidermis. J. Exp. Bot. 58: 2717-2731.
24. Kannangara R, Branigan C, Liu Y, Penfield T, Rao V, et al. (2007) The transcription

factor WIN1/SHN1 regulates cutin biosynthesis in *Arabidopsis thaliana*. Plant Cell 19: 1278-1294.

25. Broun P, Poindexter P, Osborne E, Jiang CZ, Riechmann JL (2004) WIN1, a transcriptional activator of epidermal wax accumulation in Arabidopsis. Proc Natl Acad Sci U S A 101: 4706-4711.
26. Aharoni A, Dixit S, Jetter R, Thoenes E, van Arkel G, et al. (2004) The SHINE clade of AP2 domain transcription factors activates wax biosynthesis, alters cuticle properties, and confers drought tolerance when overexpressed in Arabidopsis. Plant Cell 16: 2463-2480.
27. Nakano T, Suzuki K, Fujimura T, Shinshi H (2006) Genome-Wide Analysis of the ERF Gene Family in Arabidopsis and Rice. Plant Physiol. 140: 411-432.
28. Zhang JY, Broeckling CD, Sumner LW, Wang ZY (2007) Heterologous expression of two *Medicago truncatula* putative ERF transcription factor genes, WXP1 and WXP2 in Arabidopsis led to increased leaf wax accumulation and improved drought tolerance, but differential response in freezing tolerance. Plant Mol. Biol. 64: 265-278.
29. Yamaguchi-Shinozaki K, Koizumi M, Urao S, Shinozaki K (1992) Molecular cloning and characterization of 9 cDNAs for genes that are responsive to desiccation in *Arabidopsis thaliana*: Sequence analysis of one cDNA clone that encodes a putative transmembrane channel protein. Plant Cell Physiol. 33: 217-224.
30. Fujita M, Fujita Y, Maruyama K, Seki M, Hiratsu K, et al. (2004) A dehydration-induced NAC protein, RD26, is involved in a novel ABA-dependent stress-signaling pathway. Plant J. 39: 863-876.
31. Chen W, Provart NJ, Glazebrook J, Katagiri F, Chang HS, et al. (2002) Expression profile matrix of Arabidopsis transcription factor genes suggests their putative functions in response to environmental stresses. Plant Cell 14: 559-574.
32. Lorence A, Nessler CL (2007) Pathway engineering of the plant vitamin C metabolic network. In: Verpoorte R, Alfermann AW, Johnson TS, editors. Applications of Plant Metabolic Engineering. Dordrecht: Springer. pp. 197-218.
33. Taji T, Ohsumi C, Seki M, Iuchi S, Yamaguchi-Shinozaki K, et al. (2002) Important roles of drought- and cold-inducible genes for galactinol synthase in stress tolerance in *Arabidopsis thaliana*. Plant J. 29: 417-426.
34. Goyal K, Walton LJ, Tunnacliffe A (2005) LEA proteins prevent protein aggregation due to water stress. Biochem. J. 388: 151-157.
35. Ezaki B, Sasaki K, Matsumoto H, Nakashima S (2005) Functions of two genes in aluminium (Al) stress resistance: repression of oxidative damage by the *AtBCB* gene and promotion of efflux of Al ions by the *NtGDI1* gene. J. Exp. Bot. 56: 2661-2671.
36. Mishina TE, J. Z (2007) Bacterial non-host resistance: interactions of Arabidopsis with non-adapted *Pseudomonas syringae* strains. Physiol. Plant. 131: 448-461.
37. Rouet MA, Mathieu Y, Barbier-Brygoo H, Lauriere C (2006) Characterization of active oxygen-producing proteins in response to hypo-osmolarity in tobacco and Arabidopsis cell suspensions: identification of a cell wall peroxidase. J. Exp. Bot. 57: 1323-1332.
38. Hamann T, Bennett M, Mansfield J, Somerville C (2008) Identification of cell-wall stress as a hexose-dependent and osmosensitive regulator of plant responses. Plant J. 57: 1015-1026.
39. Brodersen P, Petersen M, Pike HM, Olszak B, Skov S (2002) Knockout of Arabidopsis *ACCELERATED-CELL-DEATH11* encoding a sphingosine transfer protein causes activation of programmed cell death and defense. Genes Dev. 16: 490-502.
40. Norholm MHH, Nour-Eldin HH, Brodersen P, Mundy J, Halkier BA (2006) Expression of the Arabidopsis high-affinity hexose transporter STP13 correlates with programmed cell death. FEBS Lett. 580: 2381-2387.
41. Chen ZY, Hartmann HA, Wu MJ, Friedman EJ, Chen JG, et al. (2006) Expression analysis of the *AtMLO* gene family encoding plant-specific seven-transmembrane domain proteins. Plant Mol. Biol. 60: 583-597.
42. Gadjev I, Vanderauwera S, Gechev TS, Laloi C, Minkov IN, et al. (2006) Transcriptomic footprints disclose specificity of reactive oxygen species signaling in Arabidopsis. Plant Physiol. 141: 436-445.
43. Thompson GA, Okuyama H (2000) Lipid-linked proteins of plants. Prog. Lipid Res. 39: 19-3
